# Dual activities of ACC synthase: Novel clues regarding the molecular evolution of *ACS* genes

**DOI:** 10.1126/sciadv.abg8752

**Published:** 2021-11-10

**Authors:** Chang Xu, Bowei Hao, Gongling Sun, Yuanyuan Mei, Lifang Sun, Yunmei Sun, Yibo Wang, Yongyan Zhang, Wei Zhang, Mengyuan Zhang, Yue Zhang, Dan Wang, Zihe Rao, Xin Li, Qingxi Jeffery Shen, Ning Ning Wang

**Affiliations:** 1 Department of Plant Biology and Ecology, Tianjin Key Laboratory of Protein Sciences, College of Life Sciences, Nankai University, Tianjin 300071, China.; 2 State Key Laboratory of Medicinal Chemical Biology, Frontiers Science Center for Cell Responses, College of Life Sciences, Nankai University, Tianjin 300071, China.; 3 University of Nevada, Las Vegas, NV 89154, USA.

## Abstract

Ethylene plays profound roles in plant development. The rate-limiting enzyme of ethylene biosynthesis is 1-aminocyclopropane-1-carboxylate (ACC) synthase (ACS), which is generally believed to be a single-activity enzyme evolving from aspartate aminotransferases. Here, we demonstrate that, in addition to catalyzing the conversion of *S*-adenosyl-methionine to the ethylene precursor ACC, genuine ACSs widely have C_β_-S lyase activity. Two N-terminal motifs, including a glutamine residue, are essential for conferring ACS activity to ACS-like proteins. Motif and activity analyses of ACS-like proteins from plants at different evolutionary stages suggest that the ACC-dependent pathway is uniquely developed in seed plants. A putative catalytic mechanism for the dual activities of ACSs is proposed on the basis of the crystal structure and biochemical data. These findings not only expand our current understanding of ACS functions but also provide novel insights into the evolutionary origin of *ACS* genes.

## Teaser

Seed plant ACC synthases widely have C_β_-S lyase activity, suggesting that they may be evolved from C_β_-S lyases.

## INTRODUCTION

During the evolution from aquatic algae to terrestrial gymnosperms and angiosperms, plants underwent tremendous and extremely complex changes to adapt to the challenges of the terrestrial environment. To survive and reproduce, plants must change their growth, development, and metabolism accordingly. Gaseous ethylene is a very ancient phytohormone that exerts profound effects on many aspects of plant growth and development. It is also indispensable for plants to deal with a range of biotic and abiotic stresses (*1*, *2*). Although the emergence of ethylene as a phytohormone is considered an important bridge between the changing environment and plant developmental adaptation (*3*), the origin and evolutionary history of the ethylene biosynthesis pathway in plants remain largely unknown.

In seed plants, the biosynthesis of ethylene mainly includes three important catalytic reactions (*4*). Methionine is first converted to *S*-adenosyl-methionine (SAM), which is further converted to 1-aminocyclopropane-1-carboxylic acid (ACC) by ACC synthase (ACS), and finally, ACC is oxidized to ethylene by ACC oxidase. Among these, the conversion from SAM to ACC represents the first committed and rate-limiting step of ethylene biosynthesis. The ethylene biosynthesis pathway of seed plants is often called the ACC-dependent pathway. The ACS proteins in seed plants are encoded by a multigene family. All ACS proteins contain a conserved aspartate aminotransferase (AAT)–like domain and belong to the α superfamily of pyridoxal-5′-phosphate (PLP)–dependent enzymes (*5*, *6*).

The efficiency and level of ethylene biosynthesis in seed plants are generally higher than that in nonseed plants (*7*–*9*). Unlike the well-characterized ethylene biosynthesis pathway in seed plants, the ethylene synthesis routes in nonseed plants remain elusive. Some early studies indicated that exogenous ACC treatment can promote ethylene production in some unicellular green algae (*10*–*12*) and multicellular charophytes (*1*), mosses (*13*), and ferns (*14*). However, other studies found that although ethylene emissions can be detected in the major groups of nonseed plants, exogenous ACC cannot be converted into ethylene, providing evidence for a non–ACC-dependent ethylene biosynthesis pathway in lower plants (*7*, *8*, *15*–*17*). In addition to these contrary findings, although the ancestry of *ACS* homologous genes can be traced back to the algal genome, biochemical and molecular biological analyses of ACS-like proteins in nonseed plants are very rare due to a lack of techniques in the early days. Our group previously cloned the only two *ACS*-like genes (*PpACLs*) in the moss *Physcomitrella patens* genome and found that neither of their encoded proteins had ACS activity (*9*). Of particular note, PpACL1 actually functions as a C_β_-S lyase (*9*). It is still unclear when during evolution the ACC-dependent pathway emerged and how plants acquired the genes encoding ACS enzymes that can catalyze the conversion of SAM to ACC in the “Yang cycle” (*4*), which are termed genuine ACSs herein.

In addition to ACS, the α superfamily of PLP-dependent enzymes also includes aminotransferases and carbon-sulfur lyases (C-S lyases) (*18*). A previous bioinformatics-based study proposed that *ACS* genes might originate from *plant-ACS–like* genes that come from *AATase* genes (*19*). In particular, another study reported that an apple ACS protein, MdACS1, exhibits extremely low aminotransferase activity in addition to ACS activity (*20*). Therefore, it is generally believed that plant ACSs are evolutionarily related to aminotransferases. However, neither of the above studies included C-S lyases that belong to the same superfamily. The possibility of an evolutionary relationship between ACSs and C-S lyases has long been ignored.

Here, starting with *Arabidopsis* ACS7 (AtACS7), we found that, in addition to catalyzing the formation of the ethylene precursor ACC, genuine ACSs widely have C_β_-S lyase activity. Two N-terminal motifs and a glutamine residue were found to be essential for conferring ACS activity to ACS-like proteins during the evolutionary process.

## RESULTS

### AtACS7 has ACS and C_β_-S lyase dual enzymatic activities

**Fig. 1.**
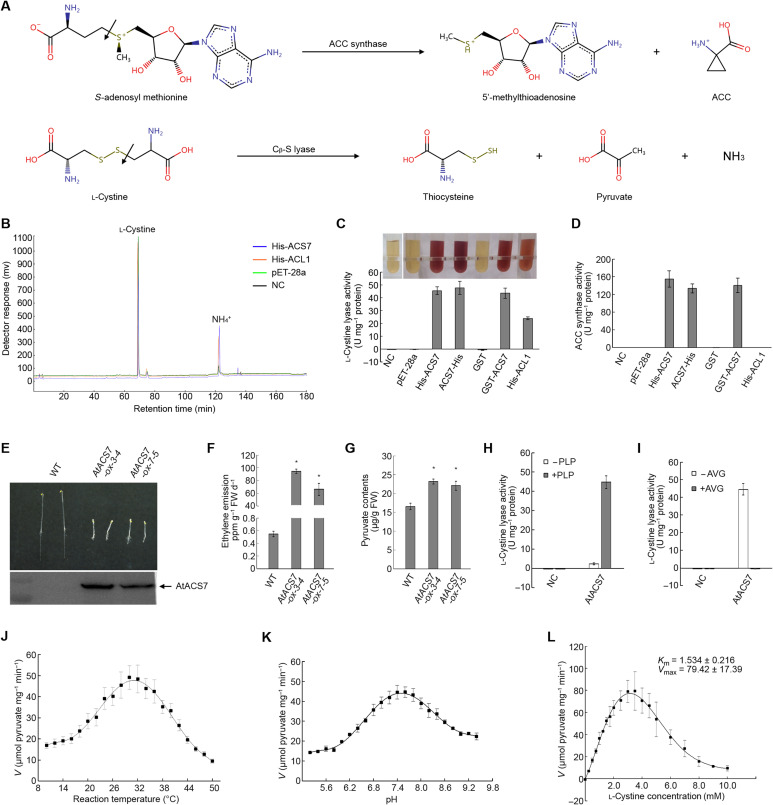
AtACS7 has ACS and C_β_-S lyase dual enzymatic activities both in vitro and in planta. (**A**) Chemical reactions catalyzed by ACSs (top) or C_β_-S lyases (bottom). (**B**) Chromatograms of NH4^+^ produced from the C_β_-S bond cleavage of l-cystine, which is catalyzed by either PpACL1 or AtACS7 in vitro. (**C**) In vitro pyruvate production assays of PpACL1- or AtACS7-containing C_β_-S lyase reaction systems. (**D**) In vitro ACS activity assays of PpACL1 and AtACS7. (**E** to **G**) The prominent accumulation of AtACS7 protein resulted in a strong triple-response phenotype, an elevated ethylene production, and a significant increase in the content of pyruvate in the etiolated seedlings of two independent *AtACS7-overexpressing* lines (*AtACS7-ox-3-4* and *AtACS7-ox-7-5*). (**H**) The C_β_-S lyase activity of purified AtACS7 was PLP dependent. (**I**) Exogenous AVG totally suppressed the C_β_-S lyase activity of purified AtACS7. (**J** and **K**) Determination of the optimal temperature and pH for C_β_-S lyase activity of purified AtACS7. (**L**) Estimation of kinetic parameters of C_β_-S lyase activity of purified AtACS7 under the optimal temperature and pH conditions. The C_β_-S lyase activities were measured by the generation of pyruvate using l-cystine as substrate. Data represent means ± SE (*n* ≥ 3, biological replicates). The number of biological replicates for each experiment is indicated in Materials and Methods. Asterisks indicate statistically significant differences based on Student’s *t* test (α = 0.01). For negative control (NC), blank buffer was used instead of purified proteins.

AtACS7 is one of the genuine ACS enzymes of ethylene biosynthesis in *Arabidopsis*, catalyzing the conversion of SAM to ACC through α,γ-elimination (Fig. 1A, top). However, when using purified AtACS7 as a control for the in vitro C_β_-S lyase activity assay of PpACL1, we unexpectedly found that AtACS7 could also catalyze the cleavage of the C_β_-S bond of the substrate l-cystine and convert it into thiocysteine, ammonia, and pyruvate (Fig. 1A, bottom).

Analysis using an amino acid analyzer revealed the production of NH_4_^+^ in both PpACL1- and AtACS7-catalyzed reactions but not in the two negative control reactions (Fig. 1B). The generation of pyruvate was examined using the 2,4-dinitrobenzene-hydrazine method. Reddish-brown colorations of 2,4-dinitrobenzene-hydrazone generated from pyruvate and 2,4-dinitrobenzene were only found in the presence of purified PpACL1 or AtACS7 with His or glutathione *S*-transferase (GST) tags. Neither the C_β_-S lyase nor the ACS activity of AtACS7 was affected by the type or position of the epitope tags (Fig. 1, C and D, and fig. S1). Together, these results showed that AtACS7 has both ACS and C_β_-S lyase activities in vitro.

To further examine whether the dual enzymatic activities of AtACS7 exist in plant cells, we measured the ethylene emissions and pyruvate contents in the *AtACS7*-overexpressing transgenic *Arabidopsis* [lines *AtACS7-ox-3-4* and *AtACS7-ox-7-5*, as used in our previous study (*21*)]. Besides the elevated ethylene production and the triple response phenotype, the higher levels of accumulated AtACS7 protein resulted in a significant increase in the content of pyruvate in both of the transgenic lines (Fig. 1, E to G). These results demonstrated that AtACS7 also has in planta C_β_-S lyase activity.

### Characterization of the C_β_-S lyase activity of AtACS7

As a PLP-dependent enzyme, it is well established that ACS requires PLP as the cofactor for its catalysis. We then tested the effects of PLP and aminoethoxyvinylglycine (AVG), a competitive inhibitor of PLP-dependent enzymes, on the C_β_-S lyase activity of purified AtACS7. The C_β_-S lyase activity of AtACS7 was also PLP dependent and could be totally suppressed by adding AVG to the reaction mixture to a final concentration of 5 μM (Fig. 1, H and I).

When using l-cystine as a substrate, AtACS7 achieved its maximal C_β_-S lyase activity at approximately 30°C (Fig. 1J), while the optimum pH was slightly basic at approximately 7.4 (Fig. 1K). Under optimal temperature and pH conditions, the enzymatic activity of AtACS7 peaked when the substrate concentration was approximately 4 mM. The maximum reaction rate reached approximately 79 μmol pyruvate mg^−1^ min^−1^, and the *K*_m_ value was determined to be 1.5 mM (Fig. 1L).

### The structures of AtACS7 and MdACS1 are nearly identical

**Fig. 2.**
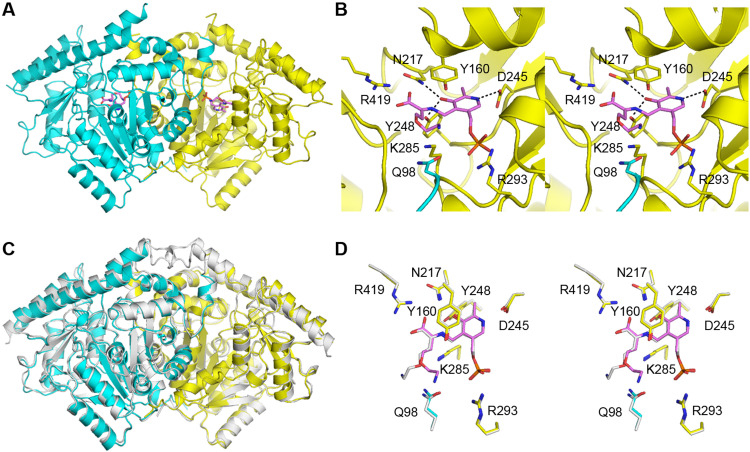
Structure of AtACS7. (**A**) Illustration of AtACS7 dimer. (**B**) The active site of AtACS7. (**C**) Superposition of the AtACS7 dimer and MdACS1 dimer. (**D**) Comparison of the active sites of AtACS7 and MdACS1. In all the panels, the two AtACS7 subunits are colored yellow (chain C) and cyan (chain D), and the PPG molecules of AtACS7 are colored violet. The contents from MdACS1 are colored white for (C) and (D). See also table S1.

To address the structural basis that may support its dual enzyme activity, we determined the crystal structure of AtACS7 in complex with pyridoxal 5′-phosphate-l-aminoethoxyvinylglycine (PPG) at 2.20-Å resolution [Protein Data Bank (PDB) code: 7DLW; statistics are summarized in table S1]. There are four AtACS7-PPG complexes that make up two dimers in each asymmetric unit. The PPG molecules showed good electron density, except for the flexible aminoethoxyvinyl groups (fig. S2A). The two dimers (chain A and B dimer and chain C and D dimer) exhibit almost identical structures, with a root mean square deviation (RMSD) of 0.76 Å, and both have the classic AAT-like folding (Fig. 2A). We used chain C and D dimer as reference for the following discussion. The structures of the two subunits are nearly the same, with an RMSD of 0.44 Å.

For the active site (Fig. 2B), Asn^217^ and Tyr^248^ both form hydrogen bonds with the O3 of PPG, and Asp^245^ forms a hydrogen bond with the N1 of PPG. Arg^293^ and Arg^419^ form salt bridges with the carboxyl group and phosphate group of PPG, respectively. Tyr^160^ exhibits parallel stacking over the pyridine ring of PPG, while the key catalytic residue Lys^285^ is located at the opposite side of the PPG from Tyr^160^. In addition to the above residues that belong to the same AtACS7 subunit, the side chain of Gln^98^ from the other subunit is protruding near the PPG molecule (Fig. 2B).

Since apple MdACS1 has been suggested to have ACS and very low aminotransferase dual activities (*20*), we subsequently compared the complex structure of AtACS7-PPG with the complex structure of MdACS1-PPG (PDB code: 1M7Y) (*22*). The overall and the active site structures of the two ACSs are almost identical (Fig. 2, C and D), with the RMSD (for the C_α_ atoms) of 0.83 Å in the overall structure comparison and 0.176 Å in the active-site residue comparison. In particular, the side-chain atoms of the above-mentioned AtACS7 active-site residues, including Q98, nearly completely overlap with their corresponding MdACS1 residues.

### The C_β_-S lyase activity of ACSs is a common phenomenon

**Fig. 3.**
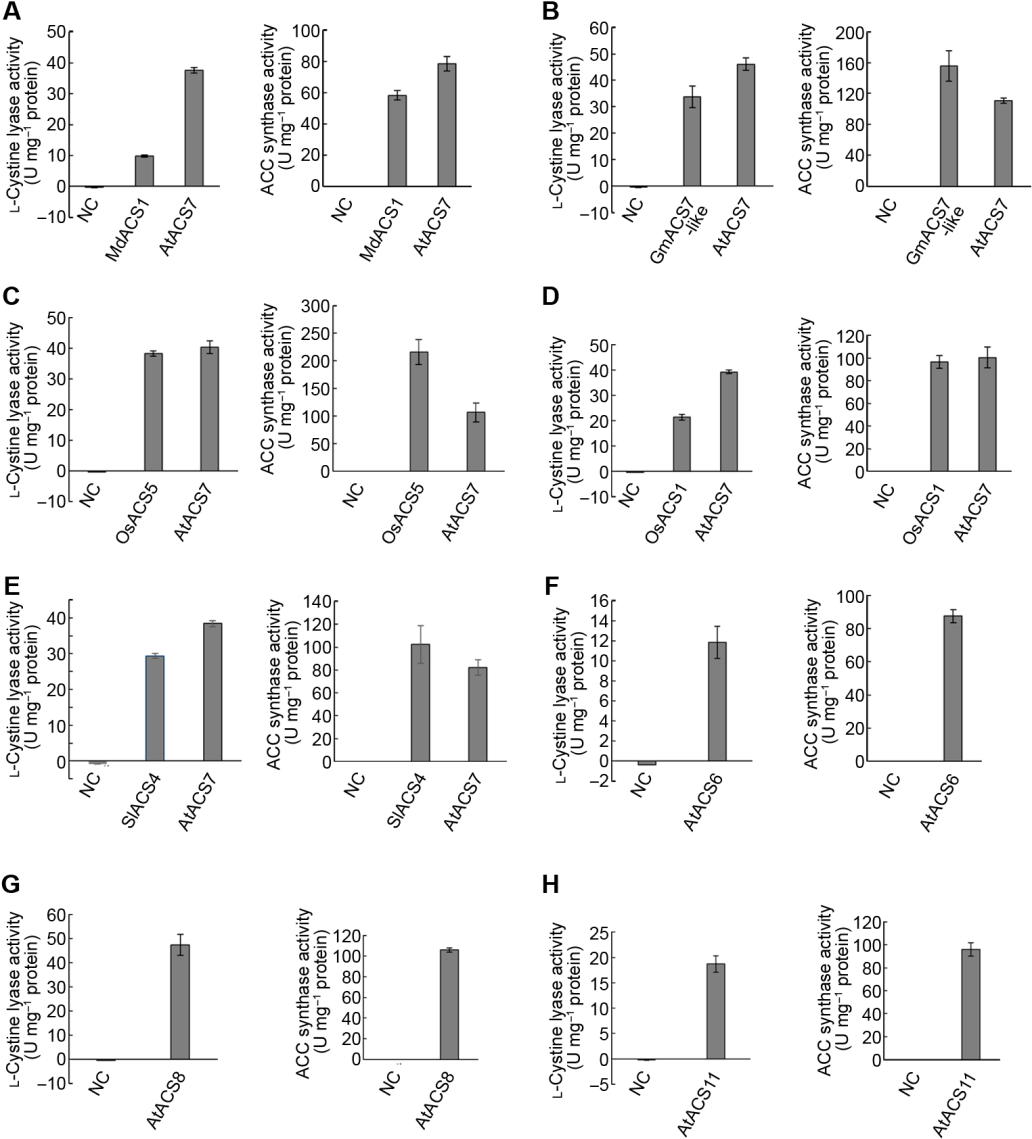
The C_β_-S lyase activity of ACS proteins may be a common phenomenon. (**A**) Apple MdACS1 has both ACS and C_β_-S lyase activities, although they are both lower than those of AtACS7. (**B** to **E**) ACS proteins in other plant species, such as GmACS7-like in soybean (Phytozome: Glyma.05G223000), OsACS5 (GenBank: X97066.1) and OsACS1 (GenBank: M96673.1) in rice, and SlACS4 in tomato (GenBank: M88487.1), have ACS and C_β_-S lyase dual activities. (**F** to **H**) ACS proteins in the model plant *Arabidopsis*, such as the type I AtACS6 (TAIR Locus: AT4G11280), type II AtACS8 [TAIR (The Arabidopsis Information Resource) Locus: AT4G37770], and AtACS11 (TAIR Locus: AT4G08040), have ACS and C_β_-S lyase activities. Purified ACS proteins were used in all in vitro assays. Data are means ± SE (*n* ≥ 3, biological replicates). The number of biological replicates for each ACS protein is indicated in Materials and Methods. AtACS7 (TAIR Locus: AT4G26200) was used as a positive control, and protein extracts from *E. coli* harboring the empty vector were used as an NC.

Since structural similarity generally indicates similarity of protein function, the extreme structural similarity between AtACS7 and MdACS1 persuaded us to purify the MdACS1 protein and examine whether it also has C_β_-S lyase activity. As expected, MdACS1 indeed had both ACS and C_β_-S lyase activities in vitro, although both were lower than AtACS7 (Fig. 3A).

ACS proteins are divided into three main groups based on their C-terminal sequences (*23*). AtACS7 is the only type III ACS in *Arabidopsis*, while MdACS1 belongs to the type II ACSs. We further examined the activities of different types of ACS proteins, such as GmACS7-like in soybean, OsACS5 and OsACS1 in rice, SlACS4 in tomato, and AtACS6, AtACS8, and AtACS11 in *Arabidopsis.* The results showed that all of them had ACS and C_β_-S lyase dual enzymatic activities (Fig. 3, B to H), suggesting that dual enzymatic activity may be a common feature of all types of ACSs in higher plants.

### Q98 plays a substantial role in ACS activity

**Fig. 4.**
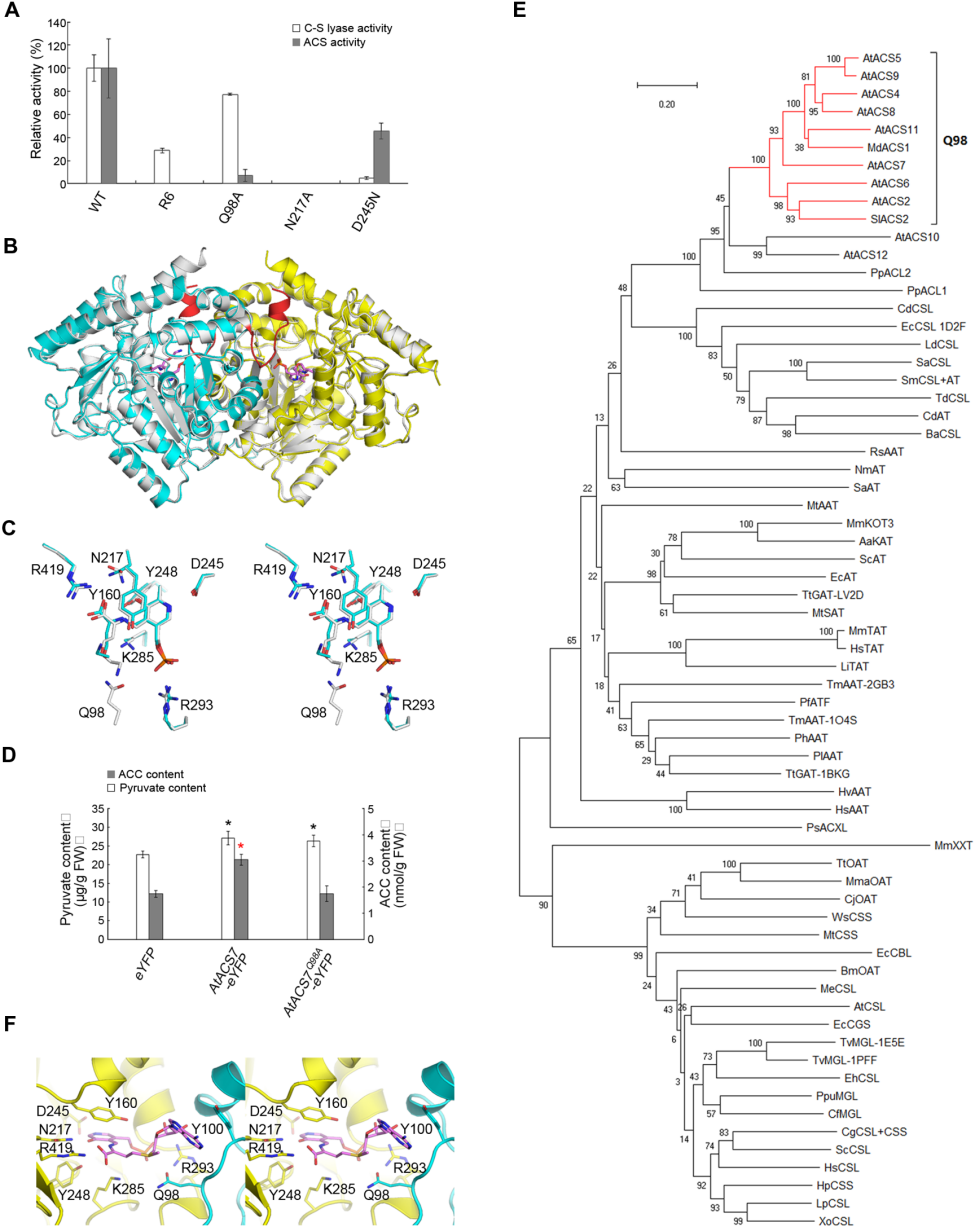
The Q98 residue plays a substantial role in conferring ACS activity. (**A**) In vitro C_β_-S lyase and ACS activity assays of purified AtACS7 mutants. The activities of wild-type AtACS7 were regarded as 100%. Data represent means ± SE (*n* ≥ 3, biological replicates). The number of biological replicates for each protein is indicated in Materials and Methods. (**B**) Superimposition of the overall structure of AtACS7-R6 and wild-type AtACS7. The two AtACS7-R6 subunits are colored yellow and cyan, respectively, and the PPG molecules are colored violet. Wild-type AtACS7 is colored white, except for the 91-to-104 region, which is colored red. (**C**) Comparison of the active sites of AtACS7-R6 (colored) and wild-type AtACS7 (white). (**D**) Determination of in planta C_β_-S lyase and ACS activities of the AtACS7^Q98A^ mutant by measuring the contents of pyruvate or ACC in the *Agrobacterium*-infiltrated tobacco leaves as described in Materials and Methods. Free *eYFP* was injected as an NC, while wild-type *AtACS7* was used as a positive control. Data represent means ± SE (*n* = 6, biological replicates). Black asterisks indicate statistically significant differences in pyruvate contents, while a red asterisk indicates statistically significant difference in ACC contents compared with the eYFP control based on Student’s *t* test (α = 0.05). (**E**) Phylogenetic analysis of functional ACS proteins, ACS-like proteins, aminotransferases, and C_β_-S lyases from a wide variety of organisms. The phylogenetic tree was constructed using the neighbor-joining method in MEGA X software. Numbers at each interior branch indicate the bootstrap values of 1000 replicates. The bar indicates a genetic distance of 0.2 cM. Detailed organisms and locus numbers or PDB IDs of all protein sequences are listed in table S2. All functional ACS proteins form a separate clade (red) containing the glutamine residue corresponding to Q98 of AtACS7. (**F**) Docking for SAM at the AtACS7 active site.

The C_β_-S lyase activities of ACS proteins in higher plants implied that ACSs have a close evolutionary relationship with C_β_-S lyases. It was previously reported that ACS proteins contain seven conserved domains (namely, boxes 1 to 7) (*24*). We swapped each of the seven boxes of *Arabidopsis* AtACS7 with *P. patens* PpACL1 and examined the enzymatic activities of these purified recombinant proteins. Among them, a recombinant named AtACS7-R6, in which the BOX2 of AtACS7 was replaced by PpACL1-BOX2, exhibited C_β_-S lyase activity but no ACS activity in the in vitro assays (Fig. 4A and fig. S3).

We subsequently determined the 2.95-Å resolution crystal structure of the AtACS7-R6 mutant in complex with PPG (PDB code: 7DLY; statistics summarized in table S1 and fig. S2B). The asymmetric unit contains one AtACS7-R6 dimer, and each AtACS7-R6 monomer forms a complex with the PPG molecule. The structures of these two subunits are nearly the same, with an RMSD of 0.12 Å. Compared with the wild-type AtACS7 dimer, the structure of the AtACS7-R6 mutant overall does not change much (with an RMSD of only 0.59 Å), except that the region replaced by PpACL1 BOX2 and adjacent region (residues 91 to 104) become disordered (Fig. 4B). Such a change shifted Q98 away from the active sites (Fig. 4C), suggesting that Q98 is crucial for the ACS activity of AtACS7, and mutation of this amino acid should diminish its ACS, but not C_β_-S lyase, activity. To test this hypothesis, we examined the activities of the AtACS7 ^Q98A^ mutant both in vitro and in planta. As expected, the mutant had high in vitro C_β_-S lyase activity but retained only 6.93% ACS activity of wild-type AtACS7 (Fig. 4A). When transiently overexpressed in tobacco leaves, wild-type *AtACS7* increased the levels of both pyruvate and ACC, while *AtACS7^Q98A^* only significantly increased the content of pyruvate but not ACC (Fig. 4D and fig. S4). These results demonstrated that in AtACS7, Q98 plays a substantial role in ACS activity but is dispensable for C_β_-S lyase activity, implying that the acquisition of this residue may be one of the key events during ACS evolution.

Phylogenetic analysis was further carried out using sequences of ACSs and a series of aminotransferases and C-S lyases with AAT-like folding from a wide variety of organisms, such as humans, mice, yeast, bacteria, protozoa, mosquitoes, barley, *Arabidopsis*, tomato, and apple (table S2). The results showed that only functional ACSs had the glutamine residue corresponding to Q98, further supporting the importance of this residue for ACS activity (Fig. 4E).

To estimate the role of Q98, SAM-aldimine was docked to the wild-type AtACS7 structure using the program AUTODOCK (*25*). The result showed that the amide group of Q98 directly approached the sulfur atom of SAM (Fig. 4F). Considering that no reaction between the amide group and sulfonium ion has been reported, we propose that Q98 functions to modulate the SAM conformation.

### Insights into the catalytic mechanism of ACSs with dual enzymatic activities

**Fig. 5.**
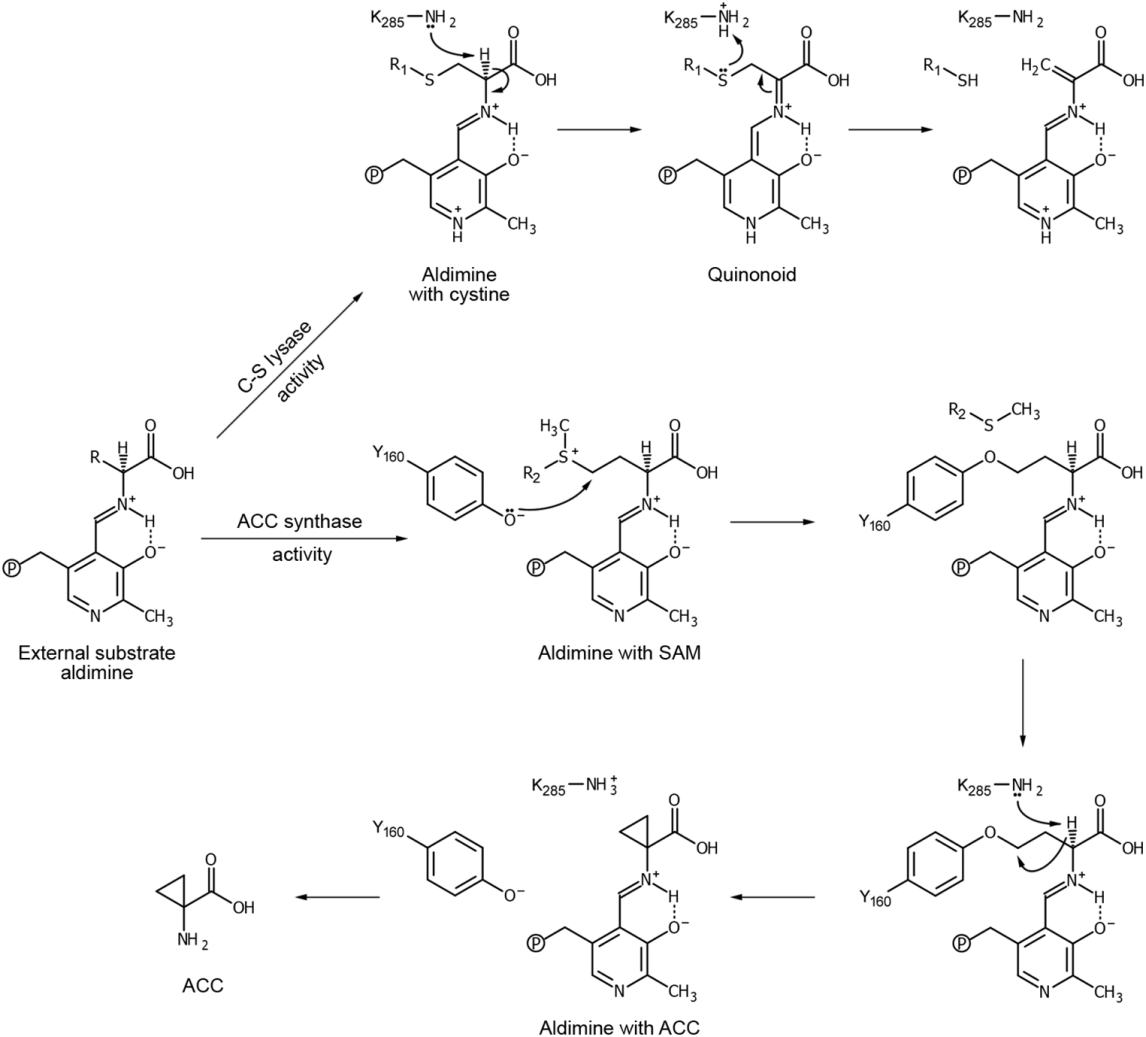
Catalytic mechanism of ACSs. Reactions catalyzed by the dual activities of ACS diverge after the formation of external substrate aldimine. For the C_β_-S lyase activity, the K285 of AtACS7 extracts the Cα proton of l-cystine and transfers it to the Sγ atom, thereby breaking the bond between Cβ and Sγ of l-cystine. A quinonoid intermediate is required in this process. Whereas for the ACS activity, a residue other than K285, probably Y160, is required to break the Cγ-Sδ bond of SAM. Following deprotonation of Cα by K285, a new covalent bond is formed between the Cα and Cγ to form ACC. In this process, the quinonoid intermediate is not essential.

In addition to AtACS7^Q98A^, we also examined the activity of the AtACS7^N217A^ and AtACS7^D245N^ mutants. The N217A mutation abolished both the ACS and C_β_-S lyase activities of AtACS7 in vitro (Fig. 4A). However, the AtACS7^D245N^ mutant showed almost no C_β_-S lyase activity but still had ACS activity, a completely opposite phenotype to the AtACS7-R6 and AtACS7^Q98A^ mutants (Fig. 4A). This observation provided insights into a novel catalytic mechanism for the ACS and C_β_-S lyase dual activities of ACS proteins (Fig. 5).

For all PLP-dependent AAT-like enzymes, the aldehyde group of PLP first forms a Schiff base linkage with an amine acid substrate (l-cystine for C_β_-S lyase activity or SAM for ACS activity) under the mediation of the lysine residue corresponding to K285 of AtACS7, thereby generating an external aldimine (*26*–*28*). In the C_β_-S lyase activity of AtACS7, on the basis of the bacterial C_β_-S lyases (*26*, *27*), the C_α_ proton is extracted by K285, producing a quinonoid intermediate. The proton is then transferred to the S_γ_ atom of the quinonoid intermediate, which breaks the bond between C_β_ and S_γ_, resulting in the release of thiol. The remaining aldimine is later hydrolyzed to produce pyruvate and ammonia. During this process, the formation of a quinonoid intermediate is critical for the catalysis. The quinonoid formation requires the protonation of pyridine nitrogen by D245. Therefore, the D245N mutation leads to the deprotonation of the pyridine nitrogen (*28*), resulting in the abrogation of C_β_-S lyase activity, which is consistent with the results of the C_β_-S lyase activity assay (Fig. 4A).

Interestingly, the AtACS7^D245N^ mutant still retained considerable, albeit reduced, ACS activity (Fig. 4A). This is not entirely consistent with previous suggestions that the catalytic process of ACS activity also requires a quinonoid intermediate (*29*, *30*), implying that the ACS activity is less dependent on the quinonoid intermediate than the C_β_-S lyase activity. We proposed that the C_γ_-S bond of SAM in the external aldimine intermediate can be broken before C_α_ deprotonation. In this case, with the C_γ_-enzyme covalent intermediate, when C_α_ is deprotonated, the α-carbanion may covalently link to C_γ_ immediately to form ACC, without forming the quinonoid intermediate. Thus, the formation of a quinonoid intermediate is no longer necessary (Fig. 5). In this process, some residue other than K285 (possibly Y160) (*29*, *30*) is required to break the C_γ_-S bond. Therefore, compared with the C_β_-S lyase activity, which only requires K285 for covalent bond breaking or bonding, the catalytic process of ACS activity is more complicated, supporting that it is evolutionarily more recent than C_β_-S lyase activity.

### Proposed structural model of ACS-like proteins with ACS activity

**Fig. 6.**
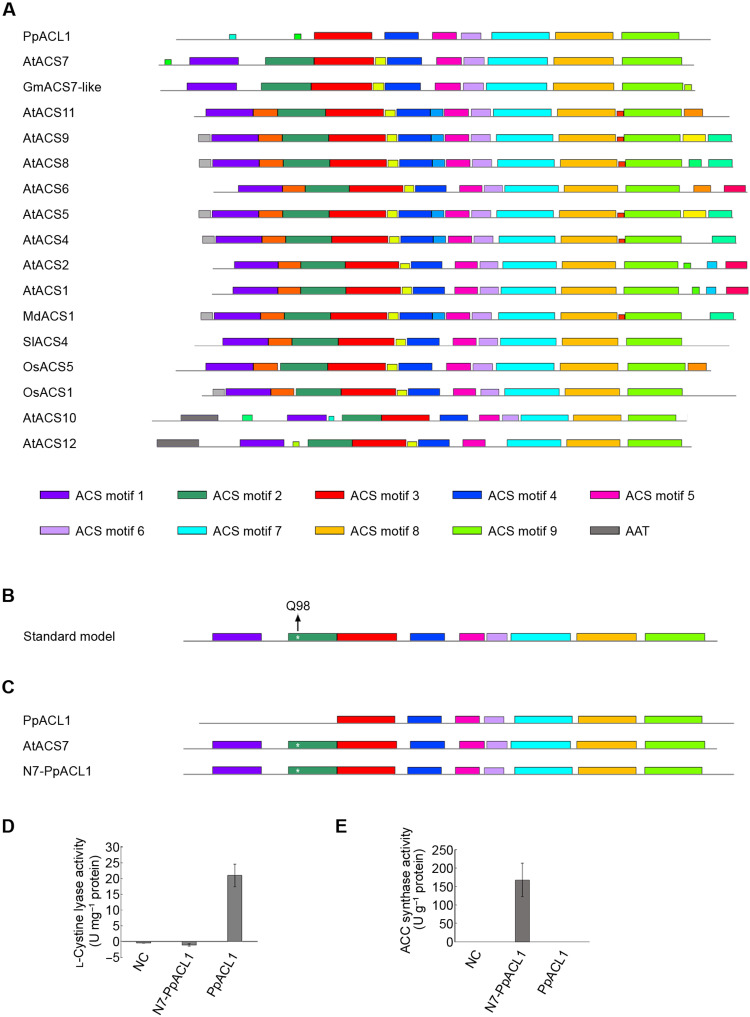
The acquisition of two N-terminal motifs is a key event for ACS activity. (**A**) MEME analysis was performed using *Arabidopsis* ACS proteins and the functionally confirmed ACS sequences from soybean, rice, and tomato in addition to *P. patens* C_β_-S lyase PpACL1 and *Arabidopsis* aminotransferases AtACS10 and AtACS12 as controls. The results revealed nine motifs (ACS motifs 1 to 9) that are collectively required in a specific order for ACS activity. The motif present only in the two *Arabidopsis* aminotransferases AtACS10 and AtACS12 was named AAT. (**B**) Proposed structural model of the genuine ACS proteins contains nine conserved ACS motifs and the glutamine residue corresponding to Q98 of AtACS7 (indicated with a star and an arrow) in the second ACS motif. (**C**) Schematic diagram of N-terminal substitution between AtACS7 and PpACL1. The N7-PpACL1 recombinant protein is composed of the N-terminal sequence of AtACS7 and the C-terminal sequence of PpACL1. The purified recombinant protein N7-PpACL1 had no C_β_-S lyase activity in vitro (**D**) but gained in vitro ACS activity (**E**). The empty vector pET28a and C_β_-S lyase PpACL1 were used as controls. Data represent means ± SE (*n* = 3, biological replicates). See also table S3.

To identify the motifs required for ACS activity, we performed web-based Multiple Expectation-maximum for Motif Elicitation (MEME) analysis using ACSs from *Arabidopsis* or ACSs that were functionally confirmed by ourselves (Fig. 3) with C_β_-S lyase PpACL1 and *Arabidopsis* aminotransferases AtACS10 and AtACS12 as controls. The results revealed highly conserved motifs among all sequences in the C terminus and variable motifs in the N terminus (Fig. 6A and table S3). Compared with those of PpACL1 and the two amino transferases, two N-terminal motifs and seven C-terminal motifs that are spatially conserved in a specific order were named ACS motifs 1 to 9. The motif that is only present in the N terminus of two aminotransferases AtACS10 and AtACS12 was named the AAT motif. Considering the key glutamine residue mentioned above, all nine ACS motifs, but excluding the AAT motif, and a glutamine residue corresponding to Q98 of AtACS7 in the second ACS motif were proposed to be collectively required for ACS activity (Fig. 6B).

### Validation of the proposed ACS model

Compared to AtACS7, the C_β_-S lyase PpACL1 has seven C-terminal ACS motifs but lacks the two N-terminal ones (Fig. 6A). We then swapped the N-terminal domain of PpACL1 with that of AtACS7 so that the recombinant protein N7-PpACL1 met our proposed model (Fig. 6C). As expected, subsequent in vitro enzymatic analysis revealed the ACS activity of N7-PpACL1 (Fig. 6, D and E). The results confirmed the model we proposed and, more importantly, demonstrated that the N terminus plays a pivotal role in conferring ACS activity to ACS-like proteins.

**Fig. 7.**
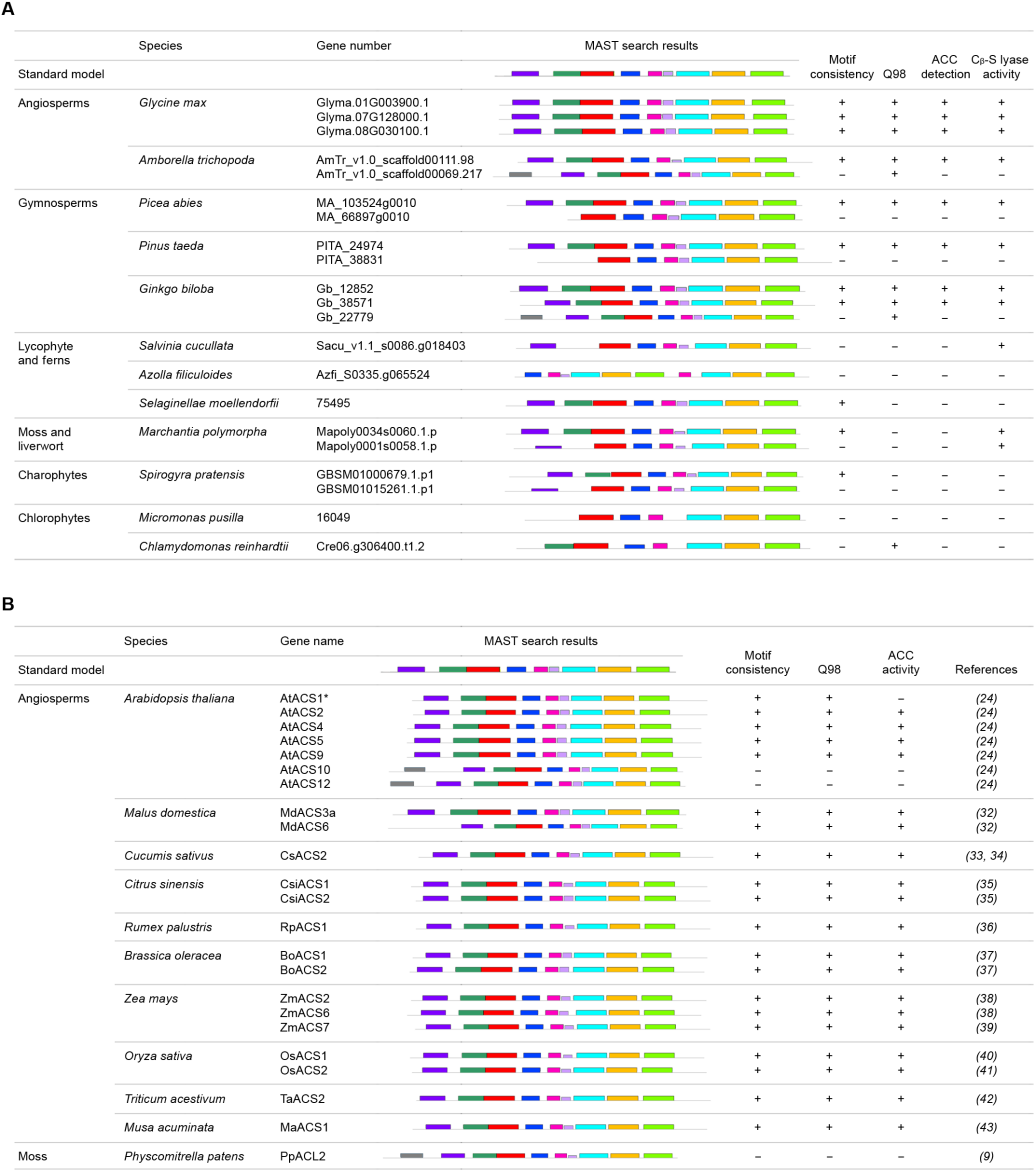
Validation of the proposed ACS model. (**A**) Validation of the proposed ACS model using ACS-like proteins from plant species ranging from chlorophytes to angiosperms. (**B**) Validation of the proposed ACS model using functionally confirmed ACS proteins in the literatures. The plus symbols (+) represent the presence of identical ACS motifs or Q98 key residue and/or exhibiting ACS or C_β_-S lyase activity, while the minus symbols (−) represent the absence of identical motifs or Q98 residue and/or no ACS or C_β_-S lyase activity detected, respectively. Data shown for ACS activity determination were based on at least four biological replicates, while those for C_β_-S lyase activity measurements were from at least three biological replicates. Raw data are presented in figs. S5 to S8. * indicates the special issue of AtACS1, which meets the model requirements but lacks a key asparagine residue as discussed in the text.

To further verify the model, we bacterially expressed 21 ACS-like proteins from plant species ranging from chlorophytes to angiosperms (fig. S5) and determined their ACS activities by measuring ACC contents in the supernatants of the transformed bacterial cultures as described in Materials and Methods (*31*). As shown in Fig. 7A and figs. S6A and S7, significant increases in ACC contents were detected in the supernatants of bacteria overexpressing ACS-like proteins that contain all nine conserved ACS motifs and the key glutamine residue corresponding to Q98 of AtACS7, for instance, Gm.01G003900.1, Gm.07G128000.1, and Gm.08G030100.1 of *Glycine max*; AmTr_v1.0_scaffold00111.98 of *Amborella trichopoda*; MA_103524g0010 of *Picea abies*; PITA_24974 of *Pinus taeda*; and Gb_12852 and Gb_38571 of *Ginkgo biloba*. In contrast, ACC contents were not detected in the bacterial cultures overexpressing the rest of the ACS-like proteins that lack any of the nine ACS motifs and/or the glutamine residue (Fig. 7A and figs. S6A and S7), further supporting the standard ACS model we proposed (Fig. 6B). These included AmTr_v1.0_scaffold00069.217 of *A. trichopoda*, MA_66897g0010 of *P. abies*, PITA_38831 of *P. taeda*, Gb_22779 of *G. biloba*, and all the tested ACS-like proteins from nonseed plant species such as lycophytes, ferns, liverworts, mosses, charophytes, and chlorophytes (Fig. 7A). The only exception was AtACS1, which met the model requirements but did not show ACS activity, as AtACS1 lacks the asparagine corresponding to N217 in AtACS7. This residue interacts with PLP and plays an essential role in all AAT-like enzymes [fig. S7 and (*24*)]. The ACS activities of several ACS-like proteins were double checked using purified recombinant GST- or His-fusion proteins to further confirm the results (fig. S6B).

In addition to these unreported ACS-like proteins, we also performed motif analysis to the ACS-like proteins whose activities have already been reported in the literature (*9*, *24*, *32*–*43*). As shown in Fig. 7B and fig. S7, all genuine ACSs meet the ACS model requirements. Those that did not show ACS activities failed to have the nine ACS motifs and/or the key Q98 residue. We also measured the C_β_-S lyase activity of ACS-like proteins listed in Fig. 7A using crude protein extracts (fig. S8A) or purified proteins (fig. S8B). We found that, except for the genuine ACSs, all tested ACS-like proteins having in vitro C_β_-S lyase activity did not meet the ACS model requirements (Fig. 7A and fig. S8), indicating that the nine motifs and the key glutamine residue are not entirely necessary for C_β_-S lyase activity.

## DISCUSSION

Although it is generally believed that all plant ACSs originated from AATase-derived plant-ACS–like proteins (*19*, *20*), our results suggested that ACSs may have a closer evolutionary relationship with C_β_-S lyases than aminotransferases. This was mainly supported by two lines of evidence: (i) Genuine ACSs with aminotransferase activity are very rare in seed plants. So far, the only example of ACC synthase with aminotransferase activity is apple MdACS1 (*20*). However, here we demonstrated that seed plant ACSs widely have considerable C_β_-S lyase activity. (ii) Neither of the two ACS-like proteins in the moss *P. patens* exhibits ACS activity, and PpACL1 functions as a C_β_-S lyase (*9*). Combined with the fact that several nonseed plant ACS–like proteins displayed C_β_-S lyase activity (Fig. 7A), it is tempting to postulate that C_β_-S lyase activity may be quite ancient and widely present in plants of different taxonomic categories. More notably, the C_β_-S lyase PpACL1 could gain ACS activity when its N terminus was replaced by that of AtACS7 (Fig. 6, C to E), confirming that ACSs are closely related to C_β_-S lyases. These findings shed new light on the molecular evolution of ACSs.

On the basis of the crystal structures and MEME analyses, we proposed that nine ACS motifs and a specific glutamine residue corresponding to Q98 of AtACS7 in the second ACS motif are required for genuine ACSs. While most of the tested ACS-like proteins from chlorophytes to angiosperms have the seven conserved ACS motifs at the C terminus, only those with a complete assembly of the nine motifs showed ACS activity, suggesting that the two ACS motifs on the N terminus are prerequisites for ACS-like proteins to gain ACS activity during evolution (Fig. 7). This also explained why the recombinant protein N7-PpACL1 could acquire ACS activity. The “natural” acquisition of the two N-terminal motifs occurred following the appearance of gymnosperm species during plant evolution (Fig. 7A). This suggests that genuine ACS activity may be unique to seed plant species.

It is controversial whether the ACS-like proteins in *Marchantia polymorpha* have ACS activity. There are two *ACS-like* genes, *Mapoly0034s0060.1* and *Mapoly0001s0058.1*, in the *Marchantia* genome. Li *et al.* (*44*) proposed that the two *Marchantia* ACS homologs, they named as MpACS1 and MpACS2, have ACS activity based on measurements of changes of ACC contents in the *Marchantia* knockout mutants and *MpACS1*- or *MpACS2*-expressing yeast cells. However, Katayose *et al.* (*45*) recently found that knockout of one or both *MpACS-like* genes did not significantly change ACC contents and ethylene production in the *Marchantia* mutants. Our results here support the findings of Katayose *et al.*: No ACC production was detected in *Escherichia coli* cell cultures expressing either of the two *Marchantia ACS–like* genes (Fig. 7A and fig. S6A). In addition, purified Mapoly0034s0060.1 or Mapoly0001s0058.1 protein failed to convert SAM to ACC in our in vitro activity assays (fig. S6B). In contrast, we found that both Mapoly0034s0060.1 and Mapoly0001s0058.1 have C_β_-S lyase activity (fig. S8B). Furthermore, both ACS-like proteins in *Marchantia* lack the key glutamine residue corresponding to the Q98 of AtACS7 (Fig. 7A and fig. S7). This residue is critical for maintaining the conformation of SAM for catalysis (Fig. 4, C and F). Mutation of this amino acid reduced the enzyme activity by more than 93% (Fig. 4, A and D). In addition, the *Marchantia* ACS–like protein Mapoly0001s0058.1 (named as MpACS2 in *44*) lacks ACS motif 2, one of the nine motifs collectively required for ACS activity (Fig. 7A). Therefore, the two ACS-like proteins in *Marchantia* do not seem to be genuine ACSs as defined here.

Endogenous ACC is detected in wild-type liverwort *M. polymorpha* (*44*, *45*). It also exists in moss and ferns (*7*, *13*). However, in this study, we found that the ACS-like proteins of liverwort *M. polymorpha* and ferns *Salvinia cucullate* and *Azolla filiculoides* do not have ACS activity (Fig. 7A and fig. S6). Hence, it is reasonable to speculate that there might be alternative pathways for ACC biosynthesis in species lacking functional, genuine ACSs as defined here.

ACS activities detected in the in vitro assays and in an *E. coli* expression system are generally consistent with those in the in planta studies (Figs. 1 and 4 and fig. S6B) (*32*–*34*, *45*). However, they are not the same as demonstrating the existence or absence of physiological activities in plants. Besides, there are also potential issues with the in planta transient expression system in tobacco. For example, expression levels of the *ACS-like* genes in transiently transformed tobacco leaves might be different from the endogenous levels expressed in a certain organ or at a certain developmental stage of plants, especially of nonseed plant species. Likewise, functions of the *ACS-like* genes of nonseed plants might be different when expressed in seed plant tobacco. Therefore, in planta functions of *ACS-like* genes and the proposed ACS model should be further tested using *ACS-like* gene knockout mutants and overexpressing lines of organisms belonging to different taxonomic classifications.

ACSs are widely known as rate-limiting enzymes in the ethylene biosynthesis pathway. Here, we showed that this enzyme also has C_β_-S lyase activity and produces pyruvate when using l-cystine as a substrate. Although only the in vivo C_β_-S lyase activity of AtACS7 was measured here (Figs. 1G and 4D), our in vitro experiments demonstrated that the C_β_-S lyase activity of ACS or ACS-like proteins simply requires PLP and cystine, both of which exist in plants. We speculate that the in vitro C_β_-S lyase activities of the tested ACS-like proteins should reflect their C_β_-S lyase activity in vivo. However, whether a given ACS-like protein has in vivo C_β_-S lyase activity needs further verification.

Pyruvate is a common metabolite that is an end product of glycolysis and an energy substrate for the mitochondrial Krebs cycle. It is also well known for its protective properties against stressful conditions in both animal and plant cells (*46*, *47*). In addition, another product of the C_β_-S lyase activity of ACSs, thiocysteine, is prone to breakage of the disulfide bond under reducing conditions and production of hydrogen sulfide, a multifunctional signaling molecule that participates in almost all aspects of plant life (*48*). Together, the discovery of the ACS and C_β_-S lyase dual enzymatic activities of ACSs in seed plants not only improves the general knowledge of the diversity of C_β_-S lyases but also greatly expands the current understanding of the biological functions of ACS proteins. However, it is also possible that ACS activity is the main function of the modern ACS and that C_β_-S lyase activity is a residual activity. Whether it has significant biological functions awaits further studies.

## MATERIALS AND METHODS

### Identification of ACS homologs

To identify ACS homologs from the sequenced genomes of land plants, lycophyte and ferns, liverworts, charophytes, and chlorophytes listed in Fig. 7, all ACS proteins of the model plant *Arabidopsis thaliana* were used to construct a hidden Markov model (HMM) profile. Subsequently, HMMER (http://hmmer.org/download.html) searches against the above-mentioned plant proteomes (table S4) were performed. All putative ACS proteins sequences obtained were evaluated and confirmed using the NCBI Conserved Domain Database (https://ncbi.nlm.nih.gov/Structure/cdd/wrpsb.cgi). Transcript sequences of the confirmed ACS protein sequences were retrieved on the basis of the locus numbers.

### Expression and purification of ACS homologous proteins

*ACS* homologous genes were either cloned from the cDNA of the corresponding plant species or chemically synthesized at the Beijing Genomics Institute. Fragments in TA cloning vectors were then transferred into pET-28a or pGEX-6p-1 expression vector by the routine digestion and ligation method. The recombinant plasmids were then sequenced for confirmation and transformed into expression host *E. coli* BL21 [Rosetta 2 (DE3) plysS]. Construction details for each gene are presented in table S5. Recombinant proteins were purified by affinity chromatography using HisTrap FF columns or Glutathione Sepharose 4 Fast Flow (GE Healthcare) columns according to the manufacturer’s instructions.

### Crystallization and structure determination

HisTrap affinity chromatography–purified AtACS7 or AtACS7-R6 mutant protein was further purified with ion exchange (Hitrap Q HP) and gel filtration (Superdex 200) chromatography. The purified protein was concentrated to 10 mg/ml in 20 mM tris (pH 8.0) and 150 mM NaCl. Before crystallization, 1 mM PLP and AVG were supplemented to the protein solutions. Using the hanging drop vapor diffusion method, AtACS7 was crystallized in 0.1 M tris (pH 8.6), 23% (w/v) polyethylene glycol (PEG)–3350, and the AtACS7-R6 mutant was crystallized in 0.1 M Hepes (pH 7.6), 6% (w/v) PEG-10000. Diffraction data were collected at SSRF (Shanghai Synchrotron Radiation Facility) synchrotron and processed with the HKL2000 (*49*) program. The structure of apple ACS (PDB code: 3PIU) was used as the searching model for AtACS7 structure determination through PHENIX (*50*), and the refined AtACS7 structure was used as the searching model for the structure of AtACS7-R6 mutant. Both structure models were refined using COOT (*51*) and PHENIX.

### ACS activity assay

The in vitro ACS activity assay using purified ACS-like proteins with His or GST tag was performed as described previously (*9*) with modifications. Briefly, 20 μg of the purified protein was pipetted into 20-ml gas chromatography (GC) vials containing 460 μl of ACS assay buffer [50 mM EPPS [N-(2-Hydroxyethyl)piperazine-N′-(3-propanesulfonic acid)] (Sigma-Aldrich) (pH 8.5), 10 μM PLP (Sigma-Aldrich), and 2 mM dithiothreitol] and 20 μl of 10 mM SAM (Sigma-Aldrich). The mixture was made to a total volume of 500 μl and then incubated at 30°C for 30 min. The amount of ACC was determined by the method in (*52*). Briefly, when the reaction was terminated, 18 drops of fresh cold mixture of 10% NaClO and saturated NaOH (1:2, v/v) was added to convert the formed ACC to ethylene. Vials were capped immediately and incubated on ice for 5 min. Ethylene emission was measured using GC (Agilent 7890A). The ACS activity was calculated as the amount of ACC converted from SAM per minute and per microgram protein according to an ACC standard curve. The protein concentration in each assay was determined by the Bradford method. Data shown for purified GmACS7-like OsACS5, OsACS1, SlACS4, and AtACS6 in Fig. 2; AtACS7-R6, AtACS7^Q98A^, AtACS7^N217A^, and AtACS7^D245N^ in Fig. 4A; N7-PpACL1 in Fig. 6E; and PITA_24974 in fig. S6B were results of three biological replicates. Data shown for ACS7 with different tags in Fig. 1D, MdACS1 and AtACS11 in Fig. 2, and Cre06.g306400.t1.2 in fig. S6B were from four biological replicates. Data obtained for AtACS8, Mapoly0001s0058.1.p, and Mapoly0034s0060.1.p were from five biological replicates. Data for OsACS6 were from six biological replicates.

Purification of some ACS-like proteins using available conventional methods is difficult. Li *et al.* (*31*) reported that ACS overexpressed in BL21 *E. coli* cells could convert the endogenous, highly prevalent SAM in the bacterial cells into ACC and subsequently secrete it into the growth medium. Following their method, we determined ACS activities of ACS-like proteins by measuring the contents of ACC accumulated in the growth medium of bacteria transformed with *ACS-like* genes. In brief, BL21 strains harboring *ACS-like* genes were grown in the LB liquid medium and then induced by isopropyl-β-d-thiogalactopyranoside (IPTG). After induction for 20 hours at 16°C, accumulations of these proteins were visualized on SDS–polyacrylamide gel electrophoresis gel. The cell cultures containing ACS-like protein with similar accumulation levels of AtACS7 were centrifuged at 10,000*g*. Aliquots of the supernatants were transferred to 20-ml GC vials and sealed with parafilm. ACC contents were indirectly determined by measuring ethylene product, as described in (*52*). Data shown for Glyma.01G003900.1, Glyma.07G128000.1, Glyma.08G030100.1, AmTr_v1.0_scaffold00111.98, MA_103524g0010, PITA_24974, Gb_12852, Gb_38571, and Mapoly0034s0060.1.p were results from three biological replicates. Data shown for AmTr_v1.0_scaffold00069.217, MA_66897g0010, Gb_22779, Mapoly0001s0058.1.p, 16049, and Cre06.g306400.t1.2 were from four biological replicates. Data for PITA_38831, Sacu_v1.1_s0086.g018403, Azfi_S0335.g065524, 75495, GBSM01000679.1.p1, and GBSM01015261.1.p1 were based on results of five biological replicates.

To measure the contents of ACC in *Agrobacterium*-infiltrated tobacco (*Nicotiana benthamiana*) leaves, *Agrobacterium tumefaciens* strain GV3101 harboring *35S:eYFP*, *35S:AtACS7^Q98A^-eYFP*, or *35S:AtACS7-eYFP* fusion gene was mixed with the strain containing P19 silencing suppressor and injected into the epidermis of the leaves of 5-week-old tobacco plants, as described previously (*53*). For each targeted leaf, half (divided by the midrib) was infiltrated with the *eYFP*-only control culture (*35S:eYFP*) and the other half with an *AtACS7* construct culture *(35S:AtACS7^Q98A^-eYFP* or *35S:AtACS7-eYFP*). After being incubated in the dark for 48 hours, five to six infiltrated leaves were pooled. ACC contents were measured as described in (*54*). Briefly, leaves were grounded in 95% ethanol and incubated at 85°C for 20 min. After centrifugation at 10,000*g* for 15 min at 4°C, the supernatant was collected and mixed with 85% ethanol by vortexing. The mixture was incubated at 70°C for 30 min and then centrifuged again at 10,000*g* for 15 min at 4°C. The supernatant was dried in a SpeedVac, and the residues were then resuspended in 1 ml of ddH_2_O for ACC quantification, as described above. Data shown here were from six biological replicates.

### Ethylene emission measurements

Ethylene emissions from 3-day-old etiolated *Arabidopsis* seedlings of the wild-type and two individual *35S:AtACS7-eGFP* transgenic lines were measured as described (*55*). Briefly, *Arabidopsis* etiolated seedlings were incubated in 12-ml vials with 3-ml liquid 1/2 MS (Murashige and Skoog) medium and grown in plant growth chamber for 24 hours (22/19°C) in darkness after being sealed with caps. Ethylene accumulated in the vials were measured by GC (Agilent 7890A), and the rate of ethylene production was expressed as nanoliter per gram of seedlings (fresh weight) per 24 hour. All experiments were performed in three biological replicates.

### Determination of C_β_-S lyase activity and kinetics

In vitro C_β_-S lyase activity was assayed using purified ACS proteins with a His or GST tag as described (*9*). Briefly, 100 μg of purified enzyme was added to 300 μl of reaction buffer (100 μM PLP, 4 mM l-cystine, and 75 mM potassium phosphate). The mixture was incubated at 30°C for 30 min before chloroform was added to denature proteins. The mixture was then centrifuged at 12,000 rpm for 10 min at 4°C. To quantify the pyruvate product, the supernatant recovered after centrifugation was mixed with 2,4-dinitrophenylhydrazine [0.1% (w/v) in 2 M HCl], and the reaction was stopped by adding 1.5 M NaOH. Then, the pyruvate content was determined by measuring the absorbance at 520 nm and comparing to a standard curve. To measure the reduction of l-cystine and production of NH_4_^+^, supernatant of the reaction mix mentioned above was purified by suction filtration (Luer syringe filter, PES (Polyethersulfone) 0.22 μm; syringe 2.5 ml) and analyzed using an amino acid analyzer (MembraPure A300, GmbH) following the manufacturer’s instructions. Data shown for the in vitro C_β_-S lyase activity of purified GmACS7-like, OsACS5, OsACS1, SlACS4, and AtACS6 in Fig. 2; AtACS7-R6, AtACS7^Q98A^, AtACS7^N217A^, and AtACS7^D245N^ in Fig. 4A; and N7-PpACL1 in Fig. 6D were from three biological replicates. Data shown for the in vitro C_β_-S lyase activity of purified ACS7 with different tags in Fig. 1D, and MdACS1 and AtACS11 in Fig. 2 were from four biological replicates. Data shown for the in vitro C_β_-S lyase activity of purified AtACS8 were from five biological replicates. Data shown for the in vitro C_β_-S lyase activity of purified OsACS6 in Fig. 2D, and Sacu_v1.1_s0086.g018403, Mapoly0001s0058.1.p, and Mapoly0034s0060.1.p in fig. S8B were results of six biological replicates.

To test the inhibitory effect of AVG, 5 μM of the inhibitor was included in the reaction mixtures. Data shown in Fig. 1I are the results of four biological replicates. The kinetic parameters *K*_m_ and *V*_max_ of AtACS7 C_β_-S lyase were calculated using the Michaelis-Menten equation. Data presented in Fig. 1 (J to L) are results of three biological replicates. Because purification of some ACS-like proteins is difficult, we measured C_β_-S lyase activities of these proteins using crude protein extracts as described in (*56*). Data shown for Glyma.07G128000.1, MA_103524g0010, PITA_24974, PITA_38831, Gb_12852, Gb_38571, Gb_22779, and Cre06.g306400.t1.2 are results from three biological replicates. Data shown for AmTr_v1.0_scaffold00111.98, AmTr_v1.0_scaffold00069.217, MA_66897g0010, 75495, and 16049 were results from four biological replicates. Data shown for Glyma.08G030100.1 were from five biological replicates. Data shown for Glyma.01G003900.1, Azfi_S0335.g065524, GBSM01015261.1.p1, and GBSM01000679.1.p1 were from six biological replicates.

In planta C_β_-S lyase activity of AtACS7^Q98A^ was determined by measuring the content of pyruvic acid in *Agrobacterium*-infiltrated tobacco (*N. benthamiana*) leaves. The same infiltration extracts prepared for ACC content measurements were used to determine the pyruvate level in each treatment. Measurements of pyruvate contents were performed following the manufacturer’s instructions (BC2000, Solarbio Life Sciences). Data shown here were based on the results of six biological replicates. For *35S:AtACS7-eGFP* stable transgenic lines and their wild-type control, 3-day-old etiolated seedlings were harvested, and pyruvate contents were quantified using the same kit (BC2000, Solarbio Life Sciences). The results presented were from three biological replicates.

### Phylogenetic analysis

Protein sequences of aminotransferases, C_β_-S lyases, and ACSs from a wide variety of organisms such as human, mouse, yeast, bacteria, protozoa, mosquito, barley, *Arabidopsis*, tomato, and apple (table S2) were aligned using MUSCLE (Multiple Sequence Alignment, https://ebi.ac.uk/Tools/msa/muscle/). The phylogenetic tree was generated using the neighbor-joining method in MEGA X software (*57*). Numbers at each interior branch indicate the bootstrap values of 1000 replicates. The bar indicates a genetic distance of 0.2 cM.

### Motif identification

Motifs of the ACS proteins were identified statistically by the web-based MEME motif finder (http://meme-suite.org/tools/meme) (*58*) with motif length set as 6 to 50 and number of motifs 25. The MAST (Motif Alignment and Search Tool) program (http://meme-suite.org/tools/mast) (*59*) was used to search protein motifs of all ACS proteins; the ACS-like and AAT proteins were also included in the analysis.

## Supplementary Materials

### This PDF file includes:

Figs. S1 to S8 Tables S1 to S5 References

## Notes

View/request a protocol for this paper from *Bio-protocol*.
