# Supplementary material for: Dual activities of ACC synthase: Novel clues regarding the molecular evolution of ACS genes
Source: Sci Adv. 2021 Nov 10;7(46):eabg8752. doi: 10.1126/sciadv.abg8752 (PMC8580319; doi:10.1126/sciadv.abg8752)
Supplement: Supplementary file 1 — Figs. S1 to S8 Tables S1 to S5 References [file sciadv.abg8752_sm.pdf]

Science Advances
AAAS

Supplementary Materials for

Dual activities of ACC synthase: Novel clues regarding the molecular evolution of ACS genes

Chang Xu, Bowei Hao, Gongling Sun, Yuanyuan Mei, Lifang Sun, Yunmei Sun, Yibo Wang, Yongyan Zhang, Wei Zhang, Mengyuan Zhang, Yue Zhang, Dan Wang, Zihe Rao, Xin Li*, Qingxi Jeffery Shen*, Ning Ning Wang*

*Corresponding author. Email: wangnn@nankai.edu.cn (N.N.W.); lix1980@nankai.edu.cn (X.L.); jeffery.shen@unlv.edu (Q.J.S.)

Published 10 November 2021, *Sci. Adv.* **7**, eabg8752 (2021)
DOI: 10.1126/sciadv.abg8752

This PDF file includes:

Figs. S1 to S8
Tables S1 to S5
References

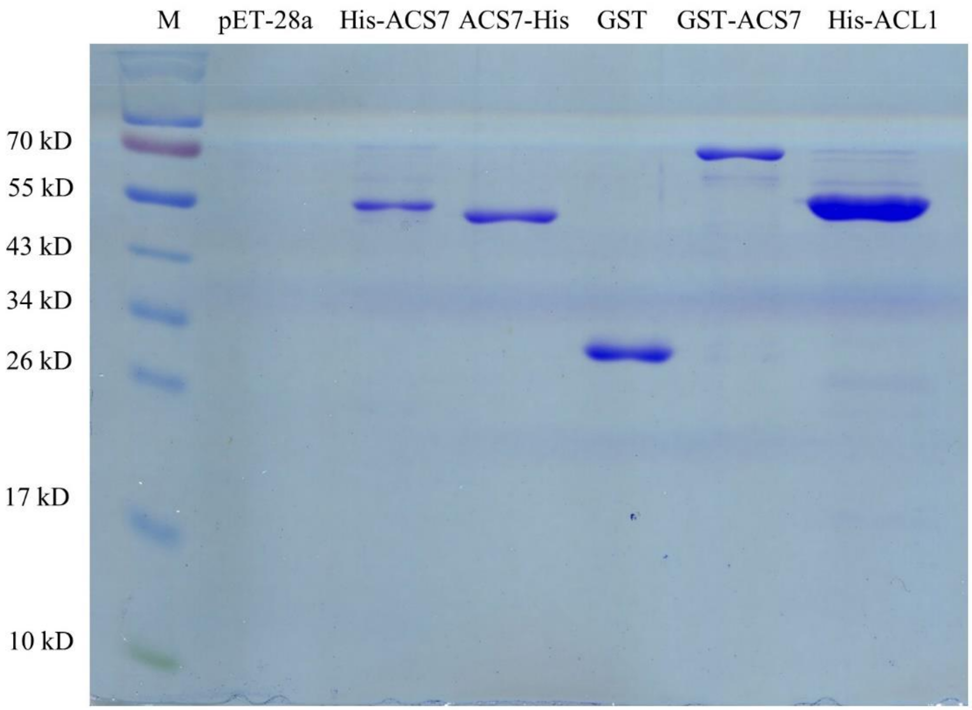

Fig. S1. Purification of PpACL1 and AtACS7 proteins with His or GST tags. Complement to Fig. 1.

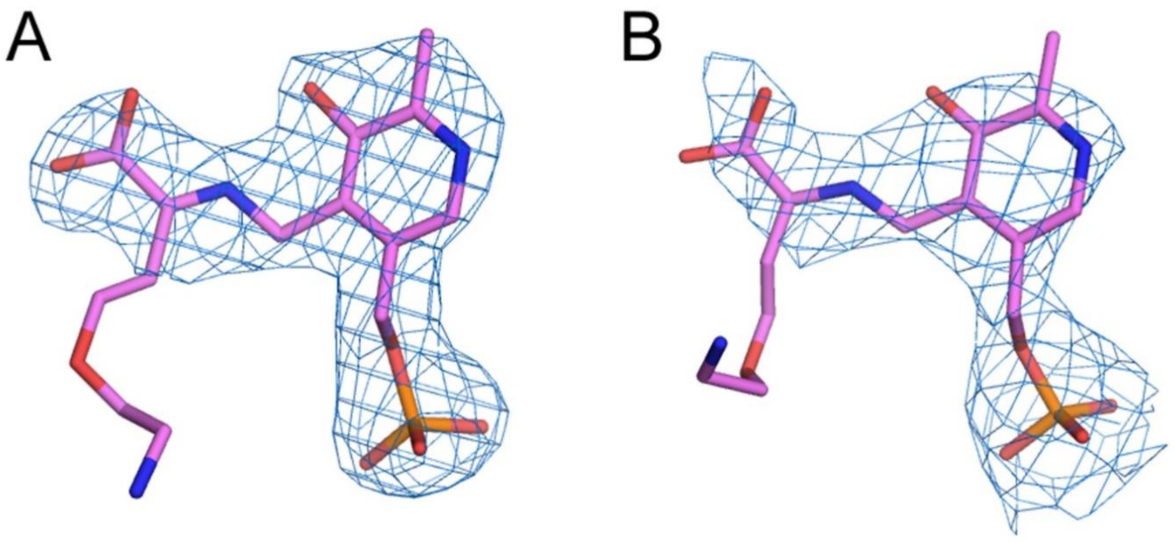

Fig. S2. Electron density of the PPG molecule. Complement to Fig. 2 and Fig. 4. The 2Fo-Fc electron density map is represented as an isomesh contoured at 1.5 sigma for each panel. (A) Wild-type AtACS7, chain C. (B) AtACS7-R6 mutant, chain A.

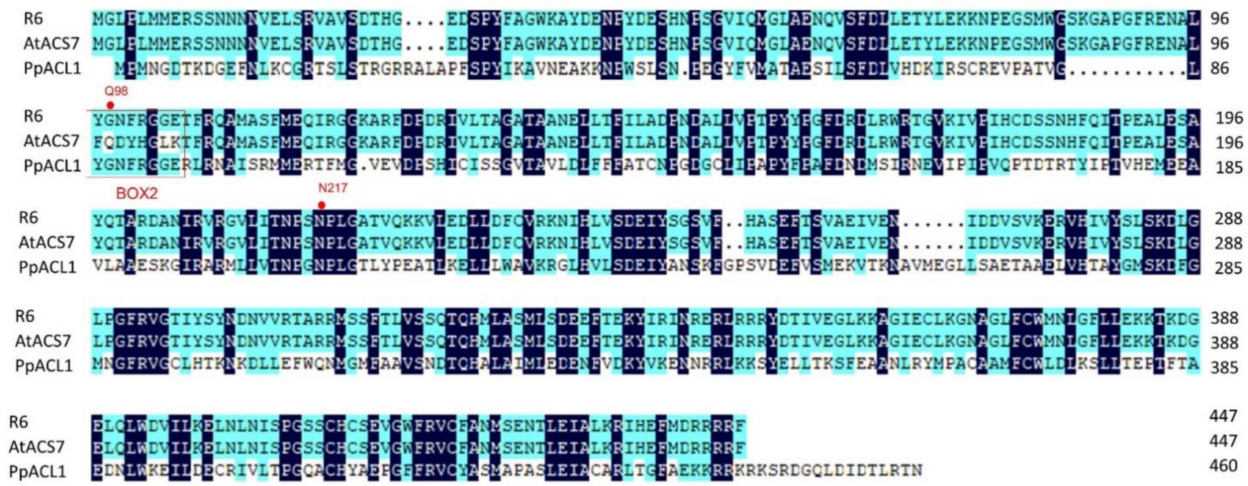

Fig. S3. Sequence alignment of AtACS7, PpACL1 and recombinant AtACS7-R6. Complement to Fig. 4. The alignment was performed using DNAMAN software with default settings. The key residues for ACS activity (Q98 and N217) are marked with red dots. The red square indicates the BOX2 region of AtACS7 and the corresponding sequences in PpACL1 and AtACS7-R6.

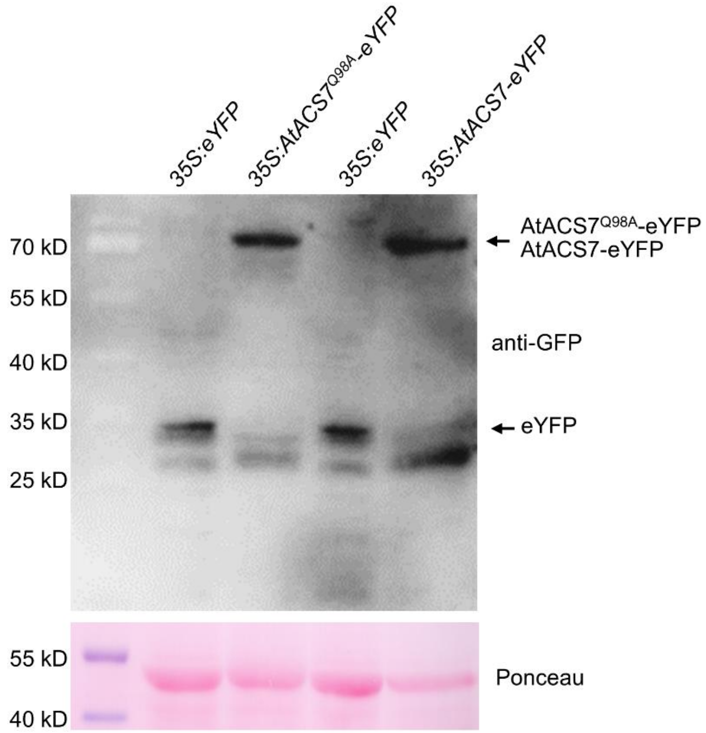

Fig. S4. Western blot showing the accumulations of eYFP, AtACS7^{Q98A}-eYFP and AtACS7-eYFP in the infiltrated tobacco leaves. Complement to Fig. 4. Arrows indicate the positions of bands of interest. Ponceau S staining of the large subunit of Rubisco was used as a loading control.

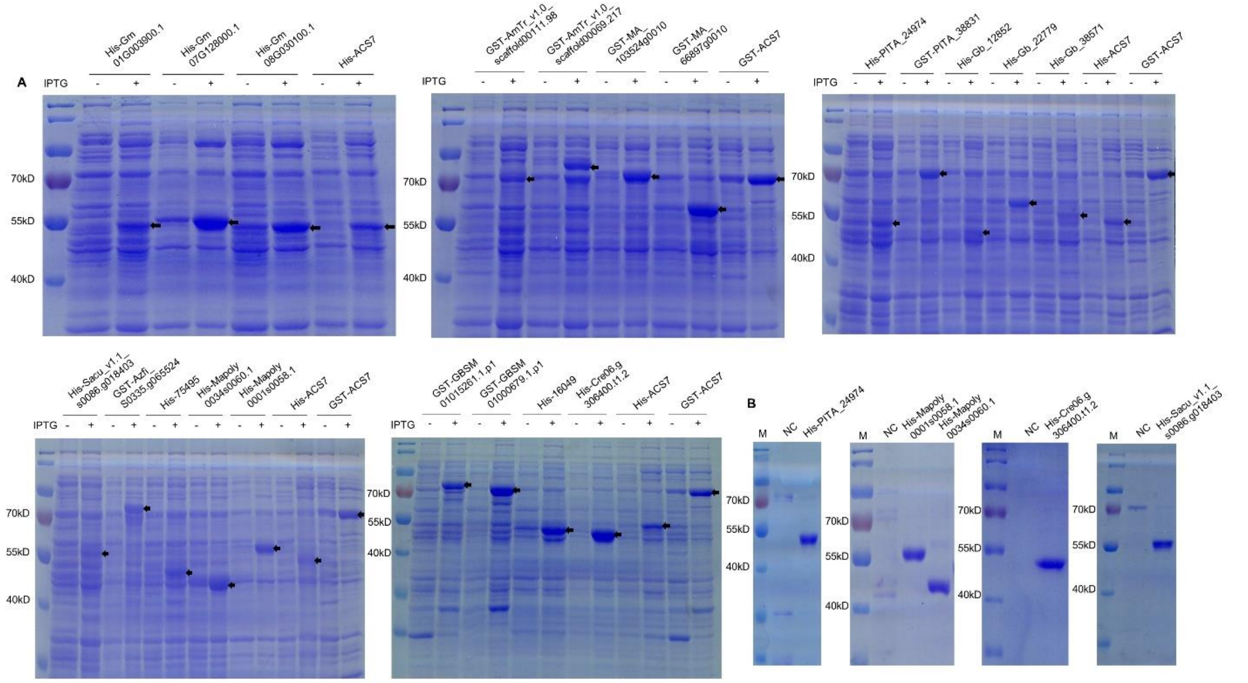

Fig. S5. Accumulations of ACS-like proteins after IPTG induction and purification. Complement to Fig. 7. (**A**) Aliquots of the supernatants of *ACS-like* gene-transformed bacterial cell cultures without or after IPTG induction were separated on SDS-PAGE gels and stained with Coomassie blue. Black arrows indicate the accumulations of the ACS-like proteins of interest. (**B**) Purified ACS-like proteins were visualized on SDS-PAGE gels and stained with Coomassie blue. NC represents the negative control, the pET-28a empty vector.

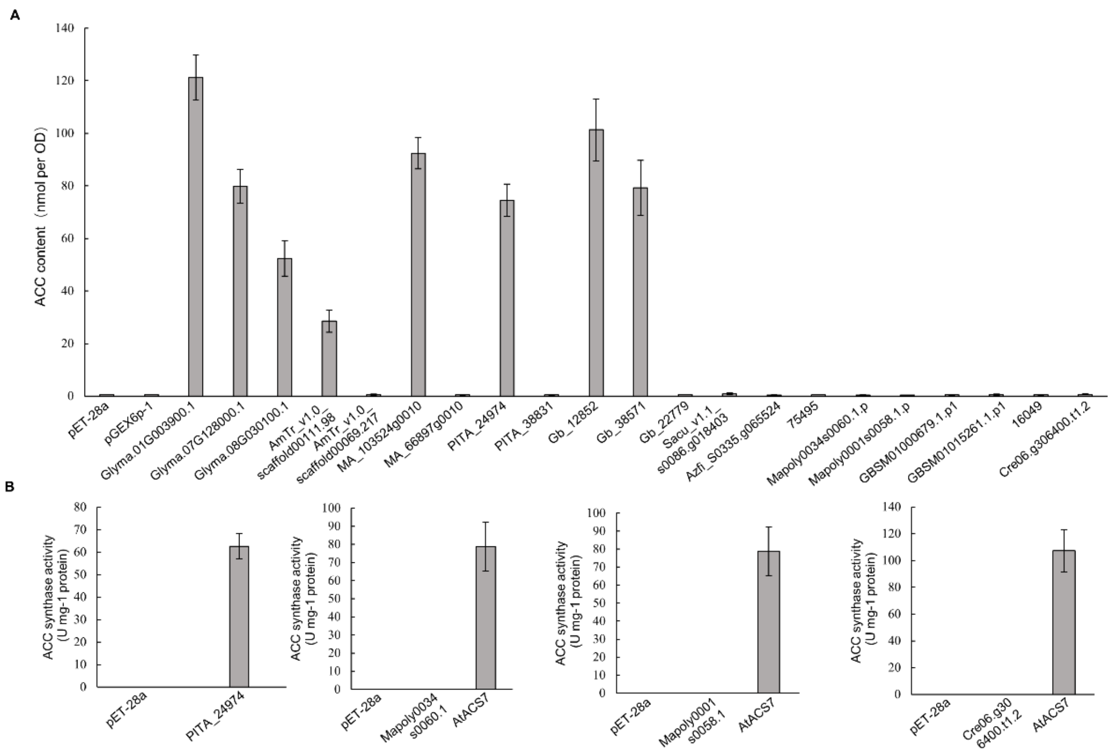

Fig. S6. *In vitro* measurements of ACS activities of ACS-like proteins from plant species ranging from chlorophytes to angiosperms. Complement to Fig 7A. (**A**) ACS activities were examined by measuring ACC contents in the supernatants of transformed bacteria as described in the Materials and Methods section. (**B**) Determinations of ACS activities of four purified ACS-like proteins. The results are consistent with those obtained by measuring ACC contents in the supernatants of the transformed bacteria shown in panel A. AtACS7 was used as a positive control while the empty vector pET-28a served as a negative control. Data represent means±SE (n⩾3, biological replicates). The number of biological replicates for each ACS-like protein is indicated in the Materials and Methods section.

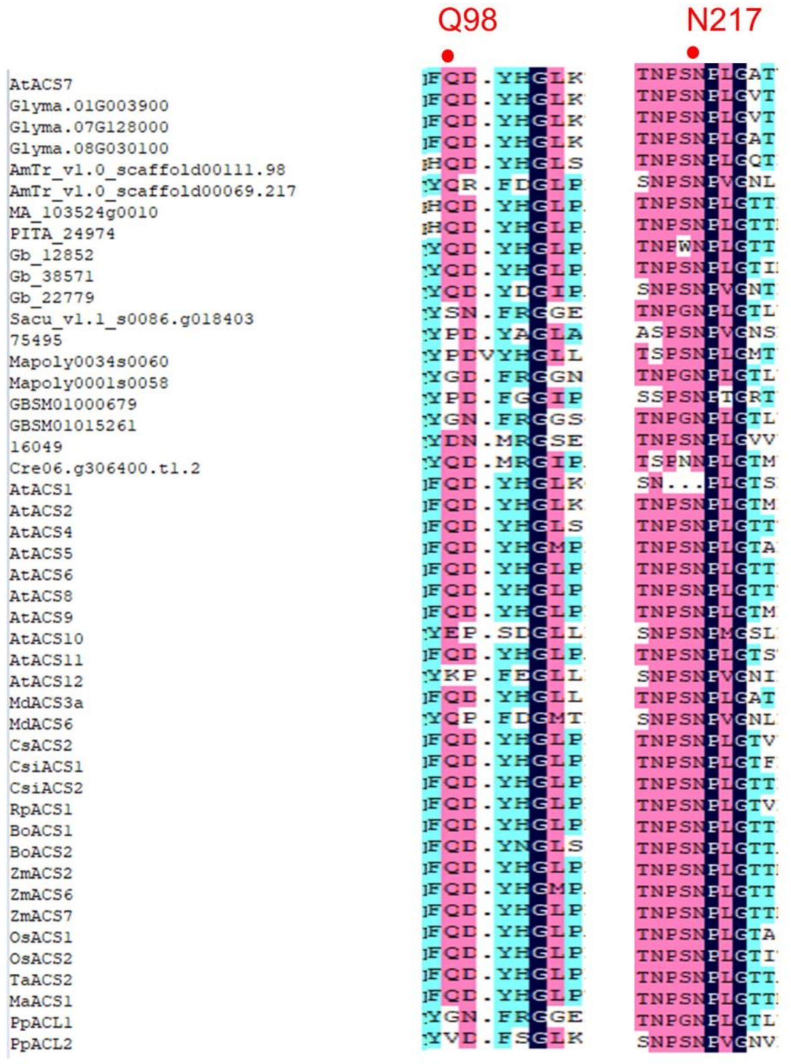

Fig. S7. Alignment of all ACS-like sequences illustrating the presence or absence of Q98 and N217 key residues. Complement to Fig 7. Sequence alignment was performed using DNAMAN software. Only regions near Q98 and N217 were shown. The two key residues are indicated with red dots.

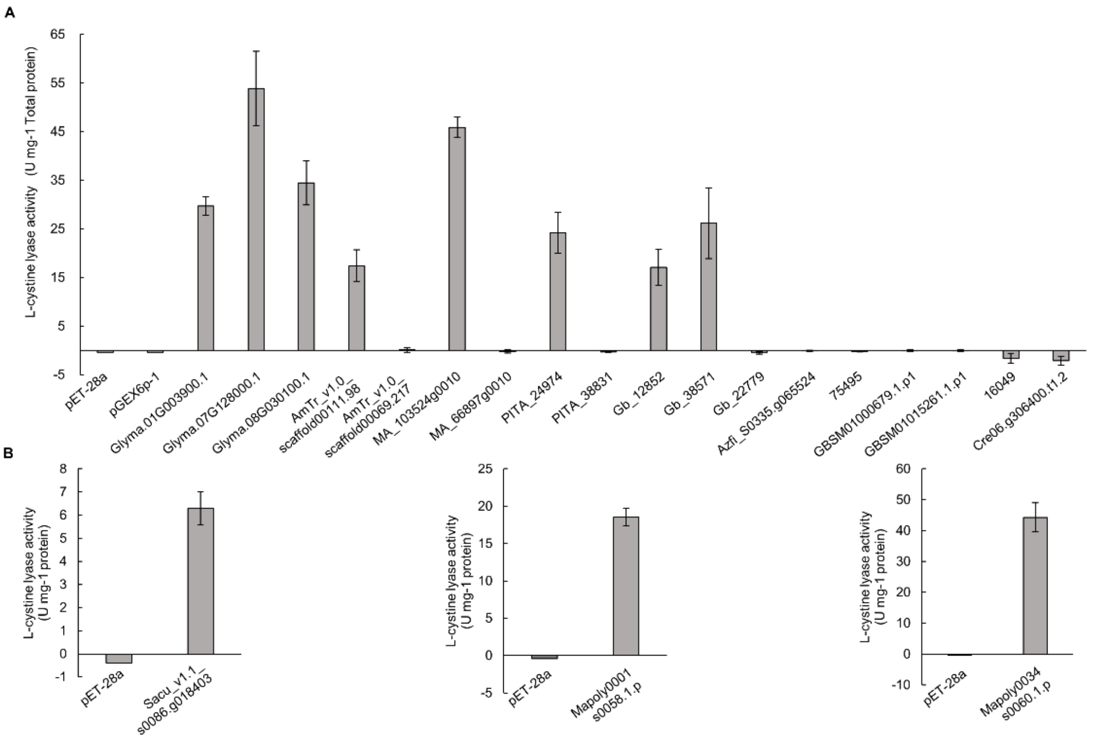

Fig. S8. ***In vitro* measurements of Cβ-S lyase activities of ACS-like proteins from plant species ranging from chlorophytes to angiosperms.** Complement to Fig. 7A. (**A**) Determinations of Cβ-S lyase activities of ACS-like proteins using crude protein extracts from the transformed *E. coli* cells. (**B**) Determinations of Cβ-S activities using purified ACS-like proteins. The empty vector pET-28a served as a negative control. Data represent means ± SE (n ⩾3, biological replicates). The number of biological replicates for each ACS-like protein is indicated in the Materials and Methods section.

Statistics	wtACS7-PPG	R6-PPG
**Data collection**		
Space group	P1	R3:H
Unit cell		
a, b, c (Å)	66.9, 69.4, 85.4	164.4, 164.4, 97.9
α, β, γ (°)	97.7, 95.0, 106.5	90, 90, 120
Resolution range (Å)	50-2.20 (2.60-2.20)	50-2.95 (3.06-2.95)
Total reflections	189,412	108,687
Unique reflections	71,649 (5,881)	20,168 (2,093)
Multiplicity	2.6 (2.6)	5.4 (6.2)
Completeness (%)	96.5 (95.1)	96.3 (99.9)
Mean I/sigma(I)	13.5 (2.1)	11.3 (1.6)
R-merge	0.066 (0.466)	0.086 (1.474)
**Refinement**		
Reflections used	71,164	20,006
Reflections for R-free	2,142	1,947
R-work	0.193	0.224
R-free	0.241	0.278
Non-hydrogen atoms	13,549	6,428
Macromolecules	13,206	6,376
Ligands	104	52
Solvent	239	N/A
Protein residues	1,658	801
RMS		
Bond length (Å)	0.008	0.011
Bond angles (°)	1.16	1.44
Ramachandran plot		
Favored (%)	97.74	92.18
Allowed (%)	2.26	7.82
Outliers (%)	0	0

Table S1. Crystallographic data collection and refinement statistics
Statistics for the highest-resolution shell are shown in parentheses.

**Organisms**	**Protein name (abbr.)**	**Locus number or PDB ID**
Arabidopsis thaliana	AtACS2/4/5/6/7/8/9/10/11/12	AT1G01480, AT2G22810, AT5G65800, AT4G11280, AT4G26200, AT4G37770, AT3G49700, AT1G62960, AT4G08040, AT5G51690
Arabidopsis thaliana	AtAAT-GAAT, AtCSL	5WMH, 1IBJ
Solanum lycopersicum	SlACS2	1IAX
Malus domestica	MdACS1	1M7Y
Physcomitrella patens	PpACL1/2	Pp1s376_33V6.1, Pp1s235_83V6.1
Escherichia coli	EcCSL, EcAT, EcCGS	1D2F, 1CL2; 1U08, 1CS1
Pyrococcus furiosus	PfATF	1XI9
Thermotoga maritima	TmATF, TmAAT	1O4S, 2GB3
Thermus thermophilus	TtGAT, TtOAT	1BKG, LV2D; 2CTZ
Pyrococcus horikoshii	PhAAT	1GD9
Phormidium lapideum	PlAAT	1J32
Mycobacterium tuberculosis	MtAAT, MtSAT, MtCSS	5YHV, 2O0R, 3NDN

Table S2. Organisms and PLP-dependent enzymes used in the phylogenetic analysis

Motifs	Width	Motif Consensus
ACS-motif-1	41	HGZDSPYFLGWKEYEKNPYDEIKNPDGIIQMGLAENQLCFD
ACS-motif-2	41	IFREJALFQDYHGLPEFKKAMADFMEEIRGNKVTFDPNRIV
ACS-motif-3	50	LTAGATSANETLMFCLADPGDAFLIPTPYYPGFDRDLKWRTGVEIVPIHC
ACS-motif-4	29	ALEEAYZQAQKRNJKVKGVLITNPSNPLG
ACS-motif-5	21	DFVTRKNIHLISDEIYSGTVF
ACS-motif-6	17	PEFVSVAEVLKDRKLEN
ACS-motif-7	50	RVHIVYSLSKDLGLPGFRVGAIYSYNDDVVSAARKMSSFGLVSSQTQHLL
ACS-motif-8	50	DKKFTENYJEENRKRLKERHKKLVEGLEEAGIECLKSNAGLFCWVDMRHL
ACS-motif-9	50	AELELWKKIVYEVKLNISPGSSCHCSEPGWFRVCFANMSEDTLEVALERJ
AAT	39	MRLIVPLRGVIQGRGGLFLGSLIPCCFFYFLQFYLKRRR

Table S3. Conserved motifs identified by MEME

In total nine conserved motifs, namely ACS-Motif 1-9, were identified as being required for ACS activity by MEME analysis. The AAT motif was only found in two aminotransferases AtACS10 and AtACS12. The width and consensus sequences were shown.

Genome	Download Site	Reference
Arabidopsis thaliana genome TAIR V10	https://phytozome.jgi.doe.gov/pz/portal.html#!bulk?org=Org_Athaliana	(*60*)
Glycine max Wm82.a2.v1	https://phytozome.jgi.doe.gov/pz/portal.html#!bulk?org=Org_Gmax	(*61*)
Amborella trichopoda genome V1.0	http://amborella.huck.psu.edu/	(*62*)
Picea abies genome V1.0	ftp://plantgenie.org/Data/ConGenIE/Picea_abies/v1.0	(*63*)
Pinus taeda genome v2.01	https://tgwebdev.cam.uchc.edu/FTP/Genomes/Pita/v2.01/	(*64*)
Ginkgo biloba genome	http://gigadb.org/dataset/100613	(*65*)
Salvinia cucullata genome	ftp://ftp.fernbase.org/	(*66*)
Azolla filiculoides genome	ftp://ftp.fernbase.org/	(*66*)
Selaginellae Moellendorfii genome V1.0	https://phytozome.jgi.doe.gov/pz/portal.html#!info?alias=Org_Smoellendorffii	(*67*)
Marchantia polymorpha genome v3.1	https://phytozome.jgi.doe.gov/pz/portal.html#!info?alias=Org_Mpolymorpha	(*68*)
Spirogyra pratensis transcriptome	https://www.ncbi.nlm.nih.gov/Traces/wgs/wgsviewer.cgi?val=GBSM01&search=GBSM01000000&display=scaffolds	(*1*)
Micromonas pusilla CCMP1545 v3.0	https://phytozome.jgi.doe.gov/pz/portal.html#!bulk?org=Org_MpusillaCCMP1545	(*69*)
Chlamydomonas reinhardtii genome	https://phytozome.jgi.doe.gov/pz/portal.html#!info?alias=Org_Creinhardtii	(*70*)

Table S4. Download sites of the genomes analyzed in this study

Species	Gene number (abbr.)	5' restriction site	3' restriction site	TA cloning vector	Expression vector
Arabidopsis thaliana	*AtACS7*	*Bam*H I	*Not* I	*pMD18-T*	*pET28a*
	AtACS6	*Bam*H I	*Not* I	*pMD18-T*	*pET28a*
	AtACS8	*Bam*H I	*Not* I	*pMD18-T*	*pET28a*
	AtACS11	*Bam*H I	*Not* I	*pMD18-T*	*pGEX-6P-1*
Glycine max	*Glyma.05G223000*	*Bam*H I	*Not* I	*pMD18-T*	*pET28a*
	Glyma.01G003900	*Eco*R I	*Not* I	*pMD18-T*	*pET28a*
	Glyma.07G128000	*Eco*R I	*Not* I	*pMD18-T*	*pET28a*
	Glyma.08G030100	*Eco*R I	*Not* I	*pMD18-T*	*pET28a*
Oryza sativa	*OsACS1*	*Bam*H I	*Not* I	*pMV*	*pGEX-6P-1*
	OsACS5	*Bam*H I	*Sal* I	*pMV*	*pET28a*
Malus domestica	*MdACS1*	*Bam*H I	*Not* I	*PUC57*	*pGEX-6P-1*
Solanum lycopersicum	*SlACS4*	*Bam*H I	*Not* I	*pQLL*	*pET28a*
Amborella trichopoda	*AmTr_v1.0_scaffold00111.98*	*Bam*H I	*Not* I	*pMV*	*pGEX-6P-1*
	AmTr_v1.0_scaffold00069.217	*Eco*R I	*Not* I	*pQLL*	*pET28a*
Picea abies	*MA_103524g0010*	*Bam*H I	*Not* I	*PUC57-Simple*	*pGEX-6P-1*
	MA_66897g0010	*Bam*H I	*Not* I	*PUC57-Simple*	*pGEX-6P-1*
Pinus taeda	*PITA_24974*	*Bam*H I	*Not* I	*PUC57-Simple*	*pET28a*
	PITA_38831	*Bam*H I	*Not* I	*PUC57-Simple*	*pGEX-6P-1*
Ginkgo biloba	*Gb_12852*	*Bam*H I	*Not* I	*PUC57-Simple*	*pET28a*
	Gb_38571	*Bam*H I	*Not* I	*PUC57-Simple*	*pET28a*
	Gb_22779	*Bam*H I	*Not* I	*PUC57-Simple*	*pET28a*
Salvinia cucullata	*Sacu_v1.1_s0086.g018403*	*Sac* I	*Not* I	*PUC57-Simple*	*pET28a*
Azolla filiculoides	*Azfi_S0335.g065524*	*Bam*H I	*Not* I	*pMV*	*pGEX-6P-1*
Selaginellae Moellendorfii	*75495*	*Bam*H I	*Not* I	*PUC57-Simple*	*pET28a*
Marchantia polymorpha	*Mapoly0034s0060.1.p*	*Bam*H I	*Not* I	*PUC57*	*pET28a*
	Mapoly0001s0058.1.p	*Eco*R I	*Not* I	*PUC57*	*pET28a*
Spirogyra pratensis	*GBSM01000679.1.p1*	*Bam*H I	*Not* I	*PUC57-Simple*	*pGEX-6P-1*
	GBSM01015261.1.p1	*Eco*R I	*Not* I	*PUC57-Simple*	*pGEX-6P-1*
Micromonas pusilla	*16049*	*Eco*R I	*Not* I	*PUC57*	*pET28a*
Chlamydomonas reinhardtii	*Cre06.g306400.t1.2*	*Eco*R I	*Hind* III	*pMD18-T*	*pET28a*

Table S5. Details of DNA constructs used in this study

REFERENCES

1. C. Ju, B. Van de Poel, E. D. Cooper, J. H. Thierer, T. R. Gibbons, C. F. Delwiche, C. Chang, Conservation of ethylene as a plant hormone over 450 million years of evolution. *Nat. Plants* **1**, 14004 (2015).

2. H. Zhao, C. C. Yin, B. Ma, S. Y. Chen, J. S. Zhang, Ethylene signaling in rice and Arabidopsis: New regulators and mechanisms. *J. Integr. Plant Biol.* **63**, 102–125 (2021).

3. M. Dubois, L. Van den Broeck, D. Inze, The pivotal role of ethylene in plant growth. *Trends Plant Sci.* **23**, 311–323 (2018).

4. S. F. Yang, N. E. Hoffman, Ethylene biosynthesis and its regulation in higher plants. *Annu. Rev. Plant. Physiol. Plant. Mol. Biol.* **35**, 155–189 (1984).

5. F. W. Alexander, E. Sandmeier, P. K. Mehta, P. Christen, Evolutionary relationships among pyridoxal-5′-phosphate-dependent enzymes. Regio-specific α, β and γ families. *Eur. J. Biochem.* **219**, 953–960 (1994).

6. P. K. Mehta, P. Christen, Homology of 1-aminocyclopropane-1-carboxylate synthase, 8-amino-7-oxononanoate synthase, 2-amino-6-caprolactam racemase, 2,2-dialkylglycine decarboxylase, glutamate-1-semialdehyde 2,1-aminomutase and isopenicillin-N-epimerase with aminotransferases. *Biochem. Biophys. Res. Commun.* **198**, 138–143 (1994).

7. J. Chernys, H. Kende, Ethylene biosynthesis in *Regnellidium diphyllum* and *Marsilea quadrifolia*. *Planta* **200**, 113–118 (1996).

8. D. J. Osborne, J. Walters, B. V. Milborrow, A. Norville, L. M. C. Stange, Special publication Evidence for a non-ACC ethylene biosynthesis pathway in lower plants. *Phytochemistry* **42**, 51–60 (1996).

9. L. Sun, H. Dong, Nasrullah, Y. Mei, N. N. Wang, Functional investigation of two *1-aminocyclopropane-1-carboxylate (ACC) synthase-like* genes in the moss *Physcomitrella patens*. *Plant Cell Rep.* **35**, 817–830 (2016).

10. T. Vanden Driessche, C. Kevers, M. Collet, T. Gaspar, Acetabularia mediterranea and ethylene: Production in relation with development, circadian rhythms in emission, and response to external application. *J. Plant Physiol.* **133**, 635–639 (1988).

11. P. Maillard, C. Thepenier, C. Gudin, Determination of an ethylene biosynthesis pathway in the unicellular green-alga, *Haematococcus pluvialis*. Relationship between growth and ethylene production. *J. Appl. Phycol.* **5**, 93–98 (1993).

12. I. Plettner, M. Steinke, G. Malin, Ethene (ethylene) production in the marine macroalga *Ulva (Enteromorpha) intestinalis* L. (Chlorophyta, Ulvophyceae): Effect of light-stress and co-production with dimethyl sulphide. *Plant Cell Environ.* **28**, 1136–1145 (2005).

13. F. Rohwer, M. Bopp, Ethylene synthesis in moss protonema. *J. Plant Physiol.* **117**, 331–338 (1985).

14. F. L. Tittle, Auxin-stimulated ethylene production in fern gametophytes and sporophytes. *Physiol. Plant.* **70**, 499–502 (1987).

15. C. Cookson, D. J. Osborne, The stimulation of cell extension by ethylene and auxin in aquatic plants. *Planta* **144**, 39–47 (1978).

16. L. M. C. Stange, D. J. Osborne, *Contrary Effects of Ethylene and ACC on Cell Growth in the Liverwort Riella Helicophylla* (Springer, 1989).

17. S.-H. Kwa, Y.-C. Wee, P. P. Kumar, Role of ethylene in the production of sporophytes from *Platycerium coronarium* (Koenig) desv. frond and rhizome pieces cultured in vitro. *J. Plant Growth Regul.* **14**, 183–189 (1995).

18. P. K. Mehta, P. Christen, The molecular evolution of pyridoxal-5′-phosphate-dependent enzymes. *Adv. Enzymol. Relat. Areas Mol. Biol.* **74**, 129–184 (2000).

19. T. C. Zhang, Q. Qiao, Y. Zhong, Detecting adaptive evolution and functional divergence in aminocyclopropane-1-carboxylate synthase (ACS) gene family. *Comput. Biol. Chem.* **38**, 10–16 (2012).

20. L. Feng, M. K. Geck, A. C. Eliot, J. F. Kirsch, Aminotransferase activity and bioinformatic analysis of 1-aminocyclopropane-1-carboxylate synthase. *Biochemistry* **39**, 15242–15249 (2000).

21. G. Sun, Y. Mei, D. Deng, L. Xiong, L. Sun, X. Zhang, Z. Wen, S. Liu, X. You, Nasrullah, D. Wang, N. N. Wang, N-Terminus-mediated degradation of ACS7 is negatively regulated by senescence signaling to allow optimal ethylene production during leaf development in *Arabidopsis*. *Front. Plant Sci.* **8**, 2066 (2017).

22. G. Capitani, D. L. McCarthy, H. Gut, M. G. Grutter, J. F. Kirsch, Apple 1-aminocyclopropane-1-carboxylate synthase in complex with the inhibitor L-aminoethoxyvinylglycine. Evidence for a ketimine intermediate. *J. Biol. Chem.* **277**, 49735–49742 (2002).

23. W. J. Lyzenga, S. L. Stone, Regulation of ethylene biosynthesis through protein degradation. *Plant Signal. Behav.* **7**, 1438–1442 (2012).

24. T. Yamagami, A. Tsuchisaka, K. Yamada, W. F. Haddon, L. A. Harden, A. Theologis, Biochemical diversity among the 1-amino-cyclopropane-1-carboxylate synthase isozymes encoded by the Arabidopsis gene family. *J. Biol. Chem.* **278**, 49102–49112 (2003).

25. G. M. Morris, R. Huey, W. Lindstrom, M. F. Sanner, R. K. Belew, D. S. Goodsell, A. J. Olson, AutoDock4 and AutoDockTools4: Automated docking with selective receptor flexibility. *J. Comput. Chem.* **30**, 2785–2791 (2009).

26. Y. Kezuka, Y. Yoshida, T. Nonaka, Structural insights into catalysis by βC-S lyase from *Streptococcus anginosus*. *Proteins* **80**, 2447–2458 (2012).

27. H. I. Krupka, R. Huber, S. C. Holt, T. Clausen, Crystal structure of cystalysin from Treponema denticola: A pyridoxal 5′-phosphate-dependent protein acting as a haemolytic enzyme. *EMBO J.* **19**, 3168–3178 (2000).

28. B. G. Caulkins, B. Bastin, C. Yang, T. J. Neubauer, R. P. Young, E. Hilario, Y. M. Huang, C. E. Chang, L. Fan, M. F. Dunn, M. J. Marsella, L. J. Mueller, Protonation states of the

tryptophan synthase internal aldimine active site from solid-state NMR spectroscopy: Direct observation of the protonated Schiff base linkage to pyridoxal-5′-phosphate. *J. Am. Chem. Soc.* **136**, 12824–12827 (2014).

29. Q. Huai, Y. Xia, Y. Chen, B. Callahan, N. Li, H. Ke, Crystal structures of 1-aminocyclopropane-1-carboxylate (ACC) synthase in complex with aminoethoxyvinylglycine and pyridoxal-5′-phosphate provide new insight into catalytic mechanisms. *J. Biol. Chem.* **276**, 38210–38216 (2001).

30. J. F. Li, L. H. Qu, N. Li, Tyr152 plays a central role in the catalysis of 1-aminocyclopropane-1-carboxylate synthase. *J. Exp. Bot.* **56**, 2203–2210 (2005).

31. N. Li, S. Huxtable, S. F. Yang, S. D. Kung, Effects of N-terminal deletions on 1-aminocyclopropane-1-carboxylate synthase activity. *FEBS Lett.* **378**, 286–290 (1996).

32. T. Li, D. Tan, Z. Liu, Z. Jiang, Y. Wei, L. Zhang, X. Li, H. Yuan, A. Wang, Apple MdACS6 regulates ethylene biosynthesis during fruit development involving ethylene-responsive factor. *Plant Cell Physiol.* **56**, 1909–1917 (2015).

33. A. Boualem, C. Troadec, I. Kovalski, M. A. Sari, R. Perl-Treves, A. Bendahmane, A conserved ethylene biosynthesis enzyme leads to andromonoecy in two cucumis species. *PLOS ONE* **4**, e6144 (2009).

34. T. Xin, Z. Zhang, S. Li, S. Zhang, Q. Li, Z. H. Zhang, S. Huang, X. Yang, Genetic regulation of ethylene dosage for cucumber fruit elongation. *Plant Cell* **31**, 1063–1076 (2019).

35. W. S. Wong, W. Ning, P. L. Xu, S. D. Kung, S. F. Yang, N. Li, Identification of two chilling-regulated 1-aminocyclopropane-1-carboxylate synthase genes from citrus (Citrus sinensis Osbeck) fruit. *Plant Mol. Biol.* **41**, 587–600 (1999).

36. I. Rieu, S. M. Cristescu, F. J. Harren, W. Huibers, L. A. Voesenek, C. Mariani, W. H. Vriezen, RP-ACS1, a flooding-induced 1-aminocyclopropane-1-carboxylate synthase gene of Rumex palustris, is involved in rhythmic ethylene production. *J. Exp. Bot.* **56**, 841–849 (2005).

37. M. Kato, T. Kamo, R. Wang, F. Nishikawa, H. Hyodo, Y. Ikoma, M. Sugiura, M. Yano, Wound-induced ethylene synthesis in stem tissue of harvested broccoli and its effect on senescence and ethylene synthesis in broccoli florets. *Postharvest Biol. Technol.* **24**, 69–78 (2002).

38. T. E. Young, R. B. Meeley, D. R. Gallie, ACC synthase expression regulates leaf performance and drought tolerance in maize. *Plant J.* **40**, 813–825 (2004).

39. H. Li, L. Wang, M. Liu, Z. Dong, Q. Li, S. Fei, H. Xiang, B. Liu, W. Jin, Maize plant architecture is regulated by the ethylene biosynthetic gene *ZmACS7*. *Plant Physiol.* **183**, 1184–1199 (2020).

40. T. I. Zarembinski, A. Theologis, Anaerobiosis and plant growth hormones induce two genes encoding 1-aminocyclopropane-1-carboxylate synthase in rice (*Oryza sativa L.*). *Mol. Biol. Cell* **4**, 363–373 (1993).

41. H. Du, N. Wu, F. Cui, L. You, X. Li, L. Xiong, A homolog of ETHYLENE OVERPRODUCER, OsETOL1, differentially modulates drought and submergence tolerance in rice. *Plant J.* **78**, 834–849 (2014).

42. K. Subramaniam, S. Abbo, P. P. Ueng, Isolation of two differentially expressed wheat ACC synthase cDNAs and the characterization of one of their genes with root-predominant expression. *Plant Mol. Biol.* **31**, 1009–1020 (1996).

43. S. R. Choudhury, S. Roy, D. N. Sengupta, A Ser/Thr protein kinase phosphorylates MA-ACS1 (Musa acuminata 1-aminocyclopropane-1-carboxylic acid synthase 1) during banana fruit ripening. *Planta* **236**, 491–511 (2012).

44. D. Li, E. Flores-Sandoval, U. Ahtesham, A. Coleman, J. M. Clay, J. L. Bowman, C. Chang, Ethylene-independent functions of the ethylene precursor ACC in Marchantia polymorpha. *Nat. Plants* **6**, 1335–1344 (2020).

45. A. Katayose, A. Kanda, Y. Kubo, T. Takahashi, H. Motose, Distinct functions of ethylene and ACC in the basal land plant marchantia polymorpha. *Plant Cell Physiol.* **62**, 858–871 (2021).

46. Y. Zhang, P. V. Taufalele, J. D. Cochran, I. Robillard-Frayne, J. M. Marx, J. Soto, A. J. Rauckhorst, F. Tayyari, A. D. Pewa, L. R. Gray, L. M. Teesch, P. Puchalska, T. R. Funari, R. McGlauflin, K. Zimmerman, W. J. Kutschke, T. Cassier, S. Hitchcock, K. Lin, K. M. Kato, J. L. Stueve, L. Haff, R. M. Weiss, J. E. Cox, J. Rutter, E. B. Taylor, P. A. Crawford, E. D. Lewandowski, C. Des Rosiers, E. D. Abel, Mitochondrial pyruvate carriers are required for myocardial stress adaptation. *Nat. Metab.* **2**, 1248–1264 (2020).

47. L. He, Y. Jing, J. Shen, X. Li, H. Liu, Z. Geng, M. Wang, Y. Li, D. Chen, J. Gao, W. Zhang, Mitochondrial pyruvate carriers prevent cadmium toxicity by sustaining the TCA cycle and glutathione synthesis. *Plant Physiol.* **180**, 198–211 (2019).

48. L. Xuan, J. Li, X. Wang, C. Wang, Crosstalk between hydrogen sulfide and other signal molecules regulates plant growth and development. *Int. J. Mol. Sci.* **21**, 4593 (2020).

49. Z. Otwinowski, W. Minor, Processing of X-ray diffraction data collected in oscillation mode. *Methods Enzymol.* **276**, 307–326 (1997).

50. P. D. Adams, P. V. Afonine, G. Bunkoczi, V. B. Chen, I. W. Davis, N. Echols, J. J. Headd, L. W. Hung, G. J. Kapral, R. W. Grosse-Kunstleve, A. J. McCoy, N. W. Moriarty, R. Oeffner, R. J. Read, D. C. Richardson, J. S. Richardson, T. C. Terwilliger, P. H. Zwart, PHENIX: A comprehensive Python-based system for macromolecular structure solution. *Acta Crystallogr. D Biol. Crystallogr.* **66**, 213–221 (2010).

51. P. Emsley, B. Lohkamp, W. G. Scott, K. Cowtan, Features and development of Coot. *Acta Crystallogr. D Biol. Crystallogr.* **66**, 486–501 (2010).

52. M. Concepcion, C. Lizada, S. F. Yang, A simple and sensitive assay for 1-aminocyclopropane-1-carboxylic acid. *Anal. Biochem.* **100**, 140–145 (1979).

53. D. Xiao, Y. Cui, F. Xu, X. Xu, G. Gao, Y. Wang, Z. Guo, D. Wang, N. N. Wang, SENESCENCE-SUPPRESSED PROTEIN PHOSPHATASE directly interacts with the cytoplasmic domain of SENESCENCE-ASSOCIATED RECEPTOR-LIKE KINASE and negatively regulates leaf senescence in Arabidopsis. *Plant Physiol.* **169**, 1275–1291 (2015).

54. D. Tudela, E. Primo-Millo, 1-Aminocyclopropane-1-carboxylic acid transported from roots to shoots promotes leaf abscission in Cleopatra mandarin (*Citrus reshni* Hort. ex Tan.) seedlings rehydrated after water stress. *Plant Physiol.* **100**, 131–137 (1992).

55. N. Li, A. K. Mattoo, Deletion of the carboxyl-terminal region of 1-aminocyclopropane-1-carboxylic acid synthase, a key protein in the biosynthesis of ethylene, results in catalytically hyperactive, monomeric enzyme. *J. Biol. Chem.* **269**, 6908–6917 (1994).

56. P. R. Jones, T. Manabe, M. Awazuhara, K. Saito, A new member of plant CS-lyases. A cystine lyase from Arabidopsis thaliana. *J. Biol. Chem.* **278**, 10291–10296 (2003).

57. S. Kumar, G. Stecher, M. Li, C. Knyaz, K. Tamura, MEGA X: Molecular evolutionary genetics analysis across computing platforms. *Mol. Biol. Evol.* **35**, 1547–1549 (2018).

58. T. L. Bailey, C. Elkan, Fitting a mixture model by expectation maximization to discover motifs in biopolymers. *Proc. Int. Conf. Intell. Syst. Mol. Biol.* **2**, 28–36 (1994).

59. T. L. Bailey, M. Gribskov, Combining evidence using p-values: Application to sequence homology searches. *Bioinformatics* **14**, 48–54 (1998).

60. P. Lamesch, T. Z. Berardini, D. Li, D. Swarbreck, C. Wilks, R. Sasidharan, R. Muller, K. Dreher, D. L. Alexander, M. Garcia-Hernandez, A. S. Karthikeyan, C. H. Lee, W. D. Nelson, L. Ploetz, S. Singh, A. Wensel, E. Huala, The Arabidopsis Information Resource (TAIR): Improved gene annotation and new tools. *Nucleic Acids Res.* **40**, D1202–1210 (2012).

61. J. Schmutz, S. B. Cannon, J. Schlueter, J. Ma, T. Mitros, W. Nelson, D. L. Hyten, Q. Song, J. J. Thelen, J. Cheng, D. Xu, U. Hellsten, G. D. May, Y. Yu, T. Sakurai, T. Umezawa, M. K. Bhattacharyya, D. Sandhu, B. Valliyodan, E. Lindquist, M. Peto, D. Grant, S. Shu, D. Goodstein, K. Barry, M. Futrell-Griggs, B. Abernathy, J. Du, Z. Tian, L. Zhu, N. Gill, T.

Joshi, M. Libault, A. Sethuraman, X. C. Zhang, K. Shinozaki, H. T. Nguyen, R. A. Wing, P. Cregan, J. Specht, J. Grimwood, D. Rokhsar, G. Stacey, R. C. Shoemaker, S. A. Jackson, Genome sequence of the palaeopolyploid soybean. *Nature* **463**, 178–183 (2010).

62. Amborella Genome Project, The *Amborella* genome and the evolution of flowering plants. *Science* **342**, 1241089 (2013).

63. B. Nystedt, N. R. Street, A. Wetterbom, A. Zuccolo, Y.-C. Lin, D. G. Scofield, F. Vezzi, N. Delhomme, S. Giacomello, A. Alexeyenko, R. Vicedomini, K. Sahlin, E. Sherwood, M. Elfstrand, L. Gramzow, K. Holmberg, J. Hällman, O. Keech, L. Klasson, M. Koriabine, M. Kucukoglu, M. Käller, J. Luthman, F. Lysholm, T. Niittylä, Å. Olson, N. Rilakovic, C. Ritland, J. A. Rosselló, J. Sena, T. Svensson, C. Talavera-López, G. Theißen, H. Tuominen, K. Vanneste, Z.-Q. Wu, B. Zhang, P. Zerbe, L. Arvestad, R. Bhalerao, J. Bohlmann, J. Bousquet, R. G. Gil, T. R. Hvidsten, P. de Jong, J. M. Kay, M. Morgante, K. Ritland, B. Sundberg, S. L. Thompson, Y. Van de Peer, B. Andersson, O. Nilsson, P. K. Ingvarsson, J. Lundeberg, S. Jansson, The Norway spruce genome sequence and conifer genome evolution. *Nature* **497**, 579–584 (2013).

64. J. L. Wegrzyn, T. Falk, E. Grau, S. Buehler, R. Ramnath, N. Herndon, Cyberinfrastructure and resources to enable an integrative approach to studying forest trees. *Evol. Appl.* **13**, 228–241 (2020).

65. R. Guan, Y. Zhao, H. Zhang, G. Fan, X. Liu, W. Zhou, C. Shi, J. Wang, W. Liu, X. Liang, Y. Fu, K. Ma, L. Zhao, F. Zhang, Z. Lu, S. M. Lee, X. Xu, J. Wang, H. Yang, C. Fu, S. Ge, W. Chen, Draft genome of the living fossil *Ginkgo biloba*. *Gigascience* **5**, 49 (2016).

66. F.-W. Li, P. Brouwer, L. Carretero-Paulet, S. Cheng, J. de Vries, P.-M. Delaux, A. Eily, N. Koppers, L.-Y. Kuo, Z. Li, M. Simenc, I. Small, E. Wafula, S. Angarita, M. S. Barker, A. Bräutigam, C. dePamphilis, S. Gould, P. S. Hosmani, Y.-M. Huang, B. Huettel, Y. Kato, X. Liu, S. Maere, R. M. Dowell, L. A. Mueller, K. G. J. Nierop, S. A. Rensing, T. Robison, C. J. Rothfels, E. M. Sigel, Y. Song, P. R. Timilsena, Y. Van de Peer, H. Wang, P. K. I. Wilhelmsson, P. G. Wolf, X. Xu, J. P. Der, H. Schluepmann, G. K.-S. Wong, K. M. Pryer,

Fern genomes elucidate land plant evolution and cyanobacterial symbioses. *Nat. Plants* **4**, 460–472 (2018).

67. J. A. Banks, T. Nishiyama, M. Hasebe, J. L. Bowman, M. Gribskov, C. dePamphilis, V. A. Albert, N. Aono, T. Aoyama, B. A. Ambrose, N. W. Ashton, M. J. Axtell, E. Barker, M. S. Barker, J. L. Bennetzen, N. D. Bonawitz, C. Chapple, C. Cheng, Luiz Gustavo Guedes Correa, M. Dacre, J. De Barry, I. Dreyer, M. Elias, E. M. Engstrom, M. Estelle, L. Feng, C. Finet, S. K. Floyd, W. B. Frommer, T. Fujita, L. Gramzow, M. Gutensohn, J. Harholt, M. Hattori, A. Heyl, T. Hirai, Y. Hiwatashi, M. Ishikawa, M. Iwata, K. G. Karol, B. Koehler, U. Kolukisaoglu, M. Kubo, T. Kurata, S. Lalonde, K. Li, Y. Li, A. Litt, E. Lyons, G. Manning, T. Maruyama, T. P. Michael, K. Mikami, S. Miyazaki, S.-i. Morinaga, T. Murata, B. Mueller-Roeber, D. R. Nelson, M. Obara, Y. Oguri, R. G. Olmstead, N. Onodera, B. L. Petersen, B. Pils, M. Prigge, S. A. Rensing, D. M. Riaño-Pachón, A. W. Roberts, Y. Sato, H. V. Scheller, B. Schulz, C. Schulz, E. V. Shakirov, N. Shibagaki, N. Shinohara, D. E. Shippen, I. Sørensen, R. Sotooka, N. Sugimoto, M. Sugita, N. Sumikawa, M. Tanurdzic, G. Theissen, P. Ulvskov, S. Wakazuki, J.-K. Weng, W. W. G. T. Willats, D. Wipf, P. G. Wolf, L. Yang, A. D. Zimmer, Q. Zhu, T. Mitros, U. Hellsten, D. Loqué, R. Otillar, A. Salamov, J. Schmutz, H. Shapiro, E. Lindquist, S. Lucas, D. Rokhsar, I. V. Grigoriev, The Selaginella genome identifies genetic changes associated with the evolution of vascular plants. *Science* **332**, 960–963 (2011).

68. J. L. Bowman, T. Kohchi, K. T. Yamato, J. Jenkins, S. Shu, K. Ishizaki, S. Yamaoka, R. Nishihama, Y. Nakamura, F. Berger, C. Adam, S. S. Aki, F. Althoff, T. Araki, M. A. Arteaga-Vazquez, S. Balasubrmanian, K. Barry, D. Bauer, C. R. Boehm, L. Briginshaw, J. Caballero-Perez, B. Catarino, F. Chen, S. Chiyoda, M. Chovatia, K. M. Davies, M. Delmans, T. Demura, T. Dierschke, L. Dolan, A. E. Dorantes-Acosta, D. M. Eklund, S. N. Florent, E. Flores-Sandoval, A. Fujiyama, H. Fukuzawa, B. Galik, D. Grimanelli, J. Grimwood, U. Grossniklaus, T. Hamada, J. Haseloff, A. J. Hetherington, A. Higo, Y. Hirakawa, H. N. Hundley, Y. Ikeda, K. Inoue, S.-I. Inoue, S. Ishida, Q. Jia, M. Kakita, T. Kanazawa, Y. Kawai, T. Kawashima, M. Kennedy, K. Kinose, T. Kinoshita, Y. Kohara, E. Koide, K. Komatsu, S. Kopischke, M. Kubo, J. Kyozuka, U. Lagercrantz, S.-S. Lin, E. Lindquist, A. M. Lipzen, C.-W. Lu, E. De Luna, R. A. Martienssen, N. Minamino, M. Mizutani, M. Mizutani,

N. Mochizuki, I. Monte, R. Mosher, H. Nagasaki, H. Nakagami, S. Naramoto, K. Nishitani, M. Ohtani, T. Okamoto, M. Okumura, J. Phillips, B. Pollak, A. Reinders, M. Rövekamp, R. Sano, S. Sawa, M. W. Schmid, M. Shirakawa, R. Solano, A. Spunde, N. Suetsugu, S. Sugano, A. Sugiyama, R. Sun, Y. Suzuki, M. Takenaka, D. Takezawa, H. Tomogane, M. Tsuzuki, T. Ueda, M. Umeda, J. M. Ward, Y. Watanabe, K. Yazaki, R. Yokoyama, Y. Yoshitake, I. Yotsui, S. Zachgo, J. Schmutz, Insights into land plant evolution garnered from the *Marchantia polymorpha* genome. *Cell* **171**, 287–304.e15 (2017).

69. A. Z. Worden, J.-H. Lee, T. Mock, P. Rouzé, M. P. Simmons, A. L. Aerts, A. E. Allen, M. L. Cuvelier, E. Derelle, M. V. Everett, E. Foulon, J. Grimwood, H. Gundlach, B. Henrissat, C. Napoli, S. M. Mc Donald, M. S. Parker, S. Rombauts, A. Salamov, P. V. Dassow, J. H. Badger, P. M. Coutinho, E. Demir, I. Dubchak, C. Gentemann, W. Eikrem, J. E. Gready, U. John, W. Lanier, E. A. Lindquist, S. Lucas, K. F. X. Mayer, H. Moreau, F. Not, R. Otillar, O. Panaud, J. Pangilinan, I. Paulsen, B. Piegu, A. Poliakov, S. Robbens, J. Schmutz, E. Toulza, T. Wyss, A. Zelensky, K. Zhou, E V. Armbrust, D. Bhattacharya, U. W. Goodenough, Y. Van de Peer, I. V. Grigoriev, Green evolution and dynamic adaptations revealed by genomes of the marine picoeukaryotes Micromonas. *Science* **324**, 268–272 (2009).

70. S. S. Merchant, S. E. Prochnik, O. Vallon, E. H. Harris, S. J. Karpowicz, G. B. Witman, A. Terry, A. Salamov, L. K. Fritz-Laylin, L. Maréchal-Drouard, W. F. Marshall, L.-H. Qu, D. R. Nelson, A. A. Sanderfoot, M. H. Spalding, V. V. Kapitonov, Q. Ren, P. Ferris, E. Lindquist, H. Shapiro, S. M. Lucas, J. Grimwood, J. Schmutz, P. Cardol, H. Cerutti, G. Chanfreau, C.-L. Chen, V. Cognat, M. T. Croft, R. Dent, S. Dutcher, E. Fernández, H. Fukuzawa, D. González-Ballester, D. González-Halphen, A. Hallmann, M. Hanikenne, M. Hippler, W. Inwood, K. Jabbari, M. Kalanon, R. Kuras, P. A. Lefebvre, S. D. Lemaire, A. V. Lobanov, M. Lohr, A. Manuell, I. Meier, L. Mets, M. Mittag, T. Mittelmeier, J. V. Moroney, J. Moseley, C. Napoli, A. M. Nedelcu, K. Niyogi, S. V. Novoselov, I. T. Paulsen, G. Pazour, S. Purton, J.-P. Ral, D. M. Riaño-Pachón, W. Riekhof, L. Rymarquis, M. Schroda, D. Stern, J. Umen, R. Willows, N. Wilson, S. L. Zimmer, J. Allmer, J. Balk, K. Bisova, C.-J. Chen, M. Elias, K. Gendler, C. Hauser, M. R. Lamb, H. Ledford, J. C. Long, J. Minagawa, M. D. Page, J. Pan, W. Pootakham, S. Roje, A. Rose, E. Stahlberg, A. M. Terauchi, P. Yang, S. Ball, C. Bowler, C. L. Dieckmann, V. N. Gladyshev, P. Green, R. Jorgensen, S. Mayfield, B.

Mueller-Roeber, S. Rajamani, R. T. Sayre, P. Brokstein, I. Dubchak, D. Goodstein, L. Hornick, Y Wayne Huang, J. Jhaveri, Y. Luo, D. Martínez, W. C. A. Ngau, B. Otillar, A. Poliakov, A. Porter, L. Szajkowski, G. Werner, K. Zhou, I. V. Grigoriev, D. S. Rokhsar, A. R. Grossman, The Chlamydomonas genome reveals the evolution of key animal and plant functions. *Science* **318**, 245–250 (2007).
